# Genetic variability of mutans streptococci revealed by wide whole-genome sequencing

**DOI:** 10.1186/1471-2164-14-430

**Published:** 2013-06-28

**Authors:** Lifu Song, Wei Wang, Georg Conrads, Anke Rheinberg, Helena Sztajer, Michael Reck, Irene Wagner-Döbler, An-Ping Zeng

**Affiliations:** 1 Institute of Bioprocess and Biosystems, Technical University Hamburg Harburg, Hamburg Harburg, Germany; 2 Division of Oral Microbiology and Immunology, Department of Operative and Preventive Dentistry & Periodontology, RWTH Aachen University, RWTH Aachen, Germany; 3 Helmholtz-Centre for Infection Research, Group Microbial Communication, Division of Microbial Pathogenesis, Inhoffenstr. 7, Braunschweig, 38124, Germany

**Keywords:** Mutans Streptococci, *Streptococcus Mutans*, *Streptococcus Ratti*, *Streptococcus Sobrinus*, Comparative Genomics, Core-genome, Pan-genome, Pathogenicity, Lactate Oxidase, Metabolic Network

## Abstract

**Background:**

Mutans streptococci are a group of bacteria significantly contributing to tooth decay. Their genetic variability is however still not well understood.

**Results:**

Genomes of 6 clinical *S*. *mutans* isolates of different origins, one isolate of *S*. *sobrinus* (DSM 20742) and one isolate of *S*. *ratti* (DSM 20564) were sequenced and comparatively analyzed. Genome alignment revealed a mosaic-like structure of genome arrangement. Genes related to pathogenicity are found to have high variations among the strains, whereas genes for oxidative stress resistance are well conserved, indicating the importance of this trait in the dental biofilm community. Analysis of genome-scale metabolic networks revealed significant differences in 42 pathways. A striking dissimilarity is the unique presence of two lactate oxidases in *S*. *sobrinus* DSM 20742, probably indicating an unusual capability of this strain in producing H_2_O_2_ and expanding its ecological niche. In addition, lactate oxidases may form with other enzymes a novel energetic pathway in *S*. *sobrinus* DSM 20742 that can remedy its deficiency in citrate utilization pathway.

Using 67 *S*. *mutans* genomes currently available including the strains sequenced in this study, we estimates the theoretical core genome size of *S*. *mutans*, and performed modeling of *S*. *mutans* pan-genome by applying different fitting models. An “open” pan-genome was inferred.

**Conclusions:**

The comparative genome analyses revealed diversities in the mutans streptococci group, especially with respect to the virulence related genes and metabolic pathways. The results are helpful for better understanding the evolution and adaptive mechanisms of these oral pathogen microorganisms and for combating them.

## Background

Traditionally and supported by 16S rRNA gene and *rnpB* gene sequence analyses, the genus *Streptococcus* is divided into several groups, with the mutans group streptococci consisting of the species *S*. *mutans*, *S*. *sobrinus*, *S*. *ratti*, *S*. *criceti*, *S*. *downei*, *S*. *macacae*, and – but controversially discussed – *S*. *ferus* (for update refer to http://www.bacterio.net/s/streptococcus.html) [1]. Mutans streptococci are considered significant contributors to the development of dental caries [2]. By attaching to the tooth surfaces and forming biofilms, they can tolerate and adapt to the harsh and rapidly changing physiological conditions of the oral cavity such as extreme acidity, fluctuation of nutrients, reactive oxygen species, and other environmental stresses [3]. They occasionally also cause bacteremia, abscesses, and infective endocarditis [4,5]. Many strains of mutans streptococci are genetically competent, i.e. they can take up DNA fragments from the environment and recombine them into their chromosome, an important mechanism for horizontal gene transfer (HGT). The ability of some bacteria to generate diversity through HGT provides a selective advantage to these microbes in their adaptation to host eco-niches and evasion of immune responses [6,7]. Due to diversities in the genetic contents between different isolates, the genome content of a single isolate does not necessarily represent the genomic potential of a certain species. With the rapid development of DNA sequencing technologies, the steadily increasing genome data enable us to dig the evolutionary and genetic information of a species from a pan-genome perspective. In 2002, the release of the genome sequence of *S*. *mutans* UA159, the first genome sequence of mutans group streptococci, has greatly helped in understanding the robustness and complexity of *S*. *mutans* as an oral and odontogenic (e.g. infective endocarditis and abscesses) pathogen [8]. Later, after the genome sequence of *S*. *mutans* NN2025 became available, a comparative genomic analysis of *S*. *mutans* NN2025 and UA159 provides insights into chromosomal shuffling and species-specific contents [9]. Recently, Cornejo *et al*. have studied the evolutionary and population genomics of *S*. *mutans* based on 57 *S*. *mutans* draft genomes and revealed a high lateral gene transfer (LGT) rate of *S*. *mutans*[10].

In this study, we determined the whole genome sequences of six *S*. *mutans* strains (5DC8, KK21, KK23, AC4446, ATCC 25175 and NCTC 11060), one *S*. *ratti* strain (DSM 20564) and one *S*. *sobrinus* strain (DSM 20742) and performed cross-comparison with the genome contents of *S*. *mutans* UA159 and NN2025, focusing on issues that are highly related to pathogenicity. The core- and pan-genome of *S*. *mutans* was analyzed by including 67 currently available *S*. *mutans* genome sequences. By constructing and comparing the genome-scale metabolic networks, the diversities in sub-networks (pathways) are systematically revealed. The results should be helpful for understanding the evolution and pathogenicity, as well as for prevention and treatment, of these very common opportunistic pathogens.

## Results and discussion

### Genome sequencing, assembly and annotation of eight mutans streptococci strains

**Figure 1:**
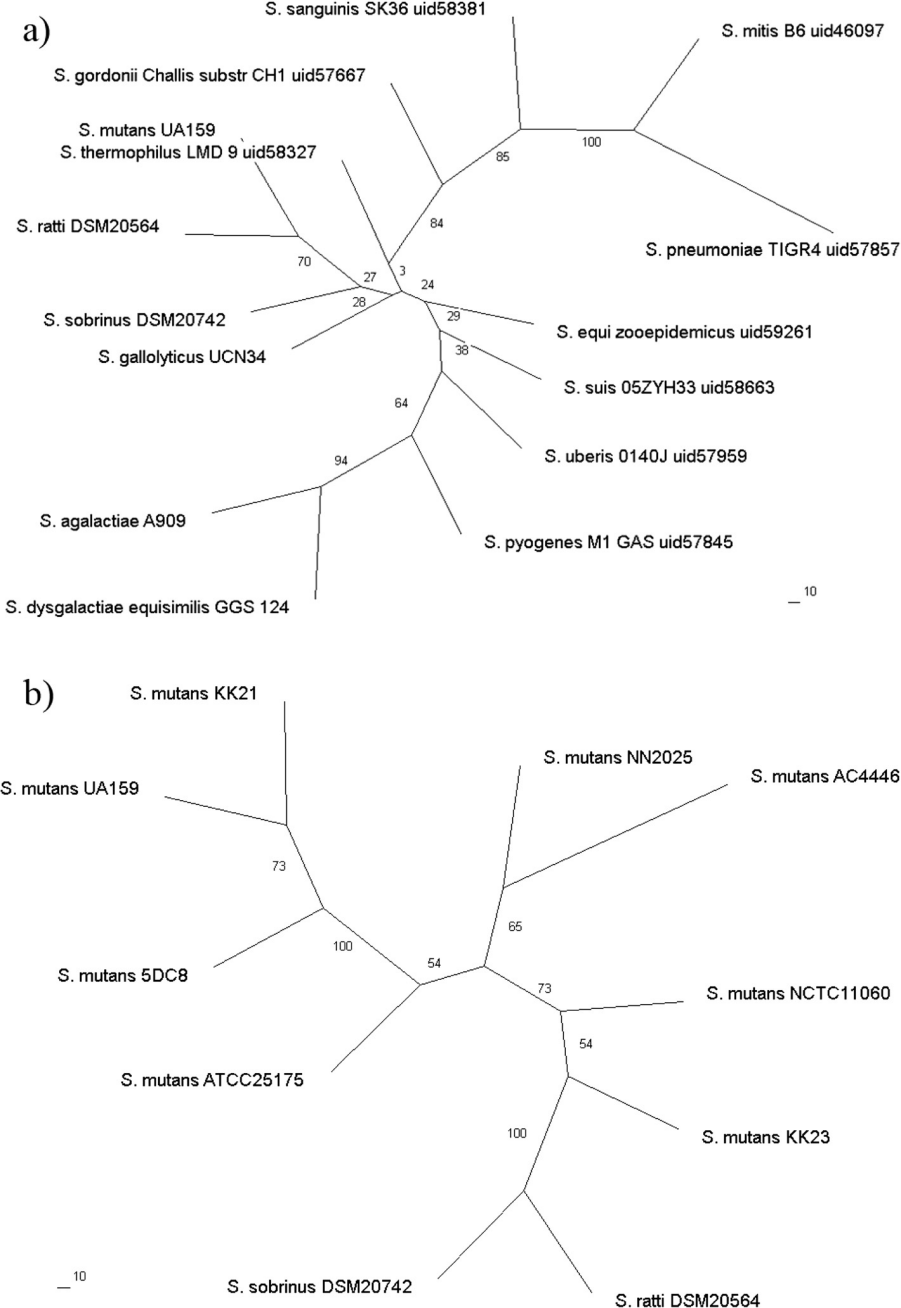
**Phylogenetic analysis of the 10 mutans streptococci strains compared in this study and their phylogenetic relationship to other *Streptococcus *species with genomes known before 01/01/2011. a)** 16S rRNA phylogenetic tree of *Streptococcus* species with genomes known before 01/01/2011 (Since the 16S rRNA sequences were almost identical between the different *S*. *mutans* strains, only UA159 is shown as representative). **b)** Phylogenetic tree of mutans streptococci constructed with the core-genome SNPs obtained by PGAP pipeline [11]. All phylogenetic trees were constructed using ClustalX and Phylip by applying the maximum likelihood (ML) method with bootstrap value set to 100. Values beside branches depict ML bootstrap support values. The scale bars in the unit of “substitution/site” are shown below the trees.

As expected, the overall genomic features of the eight *S*. *mutans* strains are more close to each other than to *S*. *ratti* and *S*. *sobrinus*. This is consistent with the results of the phylogenetic analysis, as visualized by the phylogenetic tree constructed based on 16S rRNA and core genes single-nucleotide polymorphisms (SNPs) information shown in Figure 1. An overview of the genome assemblies and annotations of the 6 *S*. *mutans* isolates as well as *S*. *ratti* DSM 20564 and *S*. *sobrinus* DSM 20742 is summarized in Table 1 in comparison with two previously sequenced *S*. *mutans* strains, namely UA159 and *S*. *mutans* NN2025. The average GC contents are in the range of low GC organisms [8]. The genome sizes are very close to each other, with the largest one from *S*. *sobrinus* DSM 20742 and the smallest one from *S*. *mutans* KK23 showing merely 5.7% differences. The total numbers of protein-coding sequences per genome are also similar among all the strains compared.

**Table 1:** **Genome assembly and annotation of eight newly sequenced mutans streptococci strains in comparison with previously sequenced *S***. ***mutans *strains UA159 and *S***. ***mutans *NN2025**

	***S. mutans *UA159**	***S. mutans *NN2025**	***S. mutans *5DC8**	***S. mutans *KK21**	***S. mutans *KK23**	***S. mutans *AC4446**	***S. mutans *ATCC 25175**	***S. mutans *NCTC 11060**	***S. ratti *DSM 20564**	***S. sobrinus *DSM 20742**
	NC_004350.2	NC_013928.1	AOBX 00000000	AOBY 00000000	AOBZ 00000000	AOCA 00000000	AOCB 00000000	AOCC 00000000	AOCD 00000000	AOCE 00000000
**Total Length**	2,030,921	2,013,587	2,010,935	2,034,586	1,976,057	2,003,537	1,999,532	2,021,202	2,037,184	2,096,203
**Contigs**	Complete	Complete	9	2	38	42	10	36	182	283
**N50 size**	Complete	Complete	354,736	1,622,660	134,323	167,413	233,425	94,580	23,860	12,417
**N90 size**	Complete	Complete	325,634	411,935	38,851	26,425	107,076	43,987	6,098	3,659
**G** + **C content**	36.82%	36.85%	36.90%	36.81%	36.68%	36.90%	36.87%	36.98%	40.29%	43.46%
**Total Genes**	2040	1975	2,004	2,031	1,933	1,999	1,982	1,982	1,995	2,057
**Protein Coding Genes**	1,960	1,895	1,924	1,949	1,907	1,919	1,903	1,915	1,965	2,032

### Chromosomal rearrangement of the *S*. *mutans strains*

**Figure 2:**
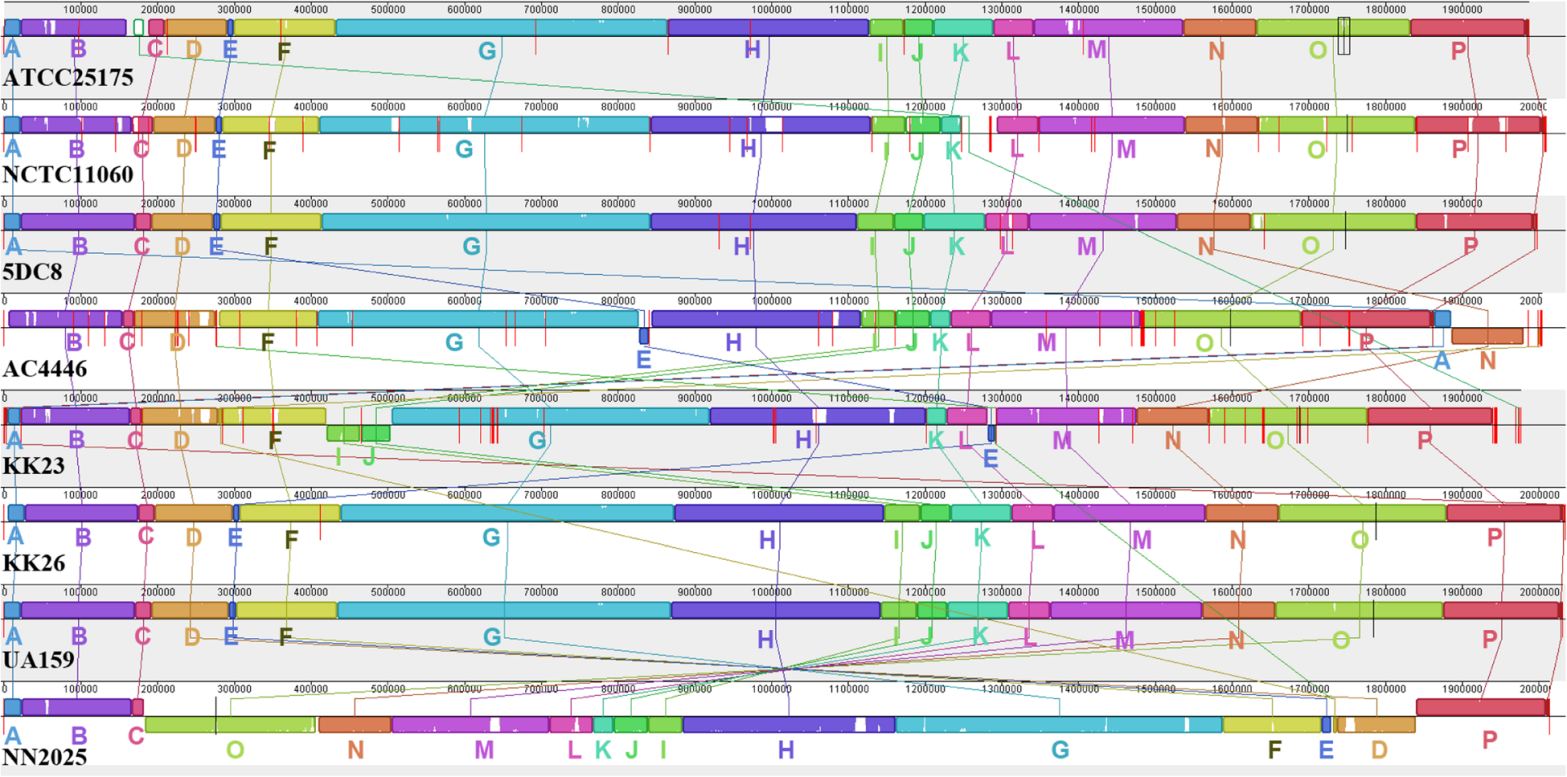
**Comparison of local collinear blocks (LCBs) of chromosomal sequences of the eight *S***. ***mutans *strains.** In total 16 local LCBs, marked as A to P, were generated and compared by applying the MAUVE software [13,14] with default settings and using strain UA159 as reference. The red vertical bars indicate contig ends. The white areas inside each LCB show regions with low similarities.

Genome rearrangements have important effects on bacterial phenotypes and the evolution of bacterial genomes [12]. A comparison of the genomes of *S*. *mutans* NN2025 and UA159 discovered a large genomic inversion across the replication axis and similar genomic variations were also confirmed among 95 clinical *S*. *mutans* isolates using long-PCR analysis [9]. In this study, genome rearrangements among the eight *S*. *mutans* strains are determined by genome alignment using the MAUVE software [13]. The results are presented in Figure 2, which shows the locally collinear blocks (LCBs) representing the landmarks, i.e. the homologous/conserved regions shared by all the input sequences in the chromosomes. A LCB is defined as a collinear (consistent) set of exactly matched subsequences (multiple maximal unique matches, namely ‘multi-MUMs’) which are shared by all the chromosomes considered, appear once in each chromosome and are bordered on both sides by mismatched nucleotides. The weight (the sum of the lengths of the included multi-MUMs) of a LCB serves as a measure of confidence that it is a true homologous region rather than a random match.

As shown in Figure 2, 16 LCBs (marked as A to P) are identified by multi-alignment of the eight *S*. *mutans* genome sequences. Compared to UA159 and NN2025, the chromosome segment represented by LCB E is reversely inserted between the LCB G and H in the strain AC4446, and between the LCB L and M in the strain KK23. This segment is related to the genomic island “*SMU*.*100*-*SMU*.*116*” of *S*. *mutans* UA159 which mainly contains sorbitol phosphotransferase system (PTS), transposase and hypothetical proteins [15]. LCB N is found to be reversed and relocated to the position between LCB A and B in the strain AC4446. A cluster of tRNA genes is found to be located downstream of LCB N. In KK23, LCB I and J are moved to position between LCB F and G. A tRNA-Gln and a tRNA-Tyr is found to be adjacent to the left of LCB I. LCB K in NCTC 11060, AC4446, KK23 and NN2025 are very similar to each other but much smaller than those of other strains (with sequence length reduced about two-thirds). The missing sequence corresponds to the genomic island TnSmu2 of *S*. *mutans* which harbors a nonribosomal peptide synthetase-polyketide synthase gene cluster responsible for the biosynthesis of pigments [16]. Using the known information about genomic islands in *S*. *mutans* UA159, additional genomic islands were found to be present/absent in the mutans streptococci strains of this study [15,17] (see Additional file 1). Furthermore, there are much more diversities as shown by the white areas inside the LCBs which show regions with low similarities. However, it should be noticed that there might be more genome rearrangements among the strains, because draft genome sequences are used in current analysis and all contigs in each genome are sorted according to the reference genome sequence of the strain UA159.

### Core- and pan-genome analysis of *S*. *mutans*

The genetic variability within species in the domain *Bacteria* is much larger than that found in other domains of life. The gene content between pairs of isolates can diverge by as much as 30% in species like *Streptococcus pneumoniae*[18]. This unexpected finding led to the introduction of the pan-genome concept, which describes the sum of genes that can be found in a given bacterial species [19,20]. The genome of any isolate is thus composed of a “core-genome” shared with all strains of this particular species, and a “dispensable genome” that accounts for the phenotypic differences between strains. The pan-genome is usually much larger than the genome of any single isolate, constituting a reservoir that could enhance the ability of many bacteria to survive in stressful environments. The pan-genome concept has important consequences for the way we understand bacterial evolution, adaptation, and population structure, as well as for more applied issues such as vaccine design or the identification of virulence genes [21]. In this study, we performed core-genome and pan-genome analyses of 67 *S*. *mutans* strains, including the eight *S*. *mutans* strains sequenced in this study and 59 *S*. *mutans* strains with genome available in NCBI till April 2013.

### Core-genome

**Figure 3:**
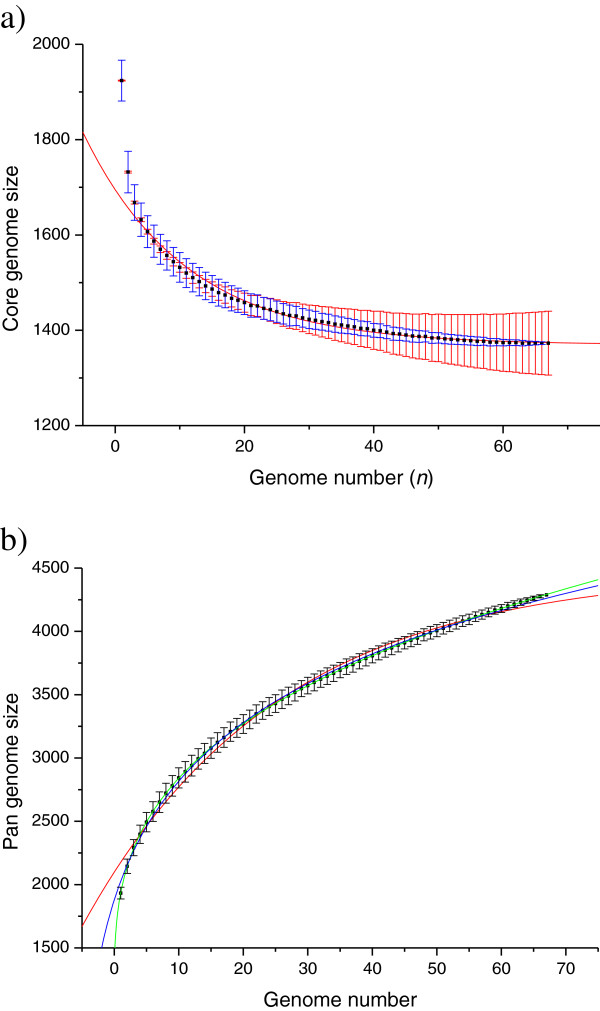
**Core and pan**-**genome model of 67 *S***. ***mutans *genomes. a)** Core-genome model of *S*. *mutans*. The core-genome size (number of common genes) is plotted as a function of the number (*n*) of genomes according to a previously proposed model Fcn=κcexp−nτc+Ω, where *K*_*c*_, τ_c_ and Ω are model parameters. Red rectangles are the medians of the core-genome sizes calculated by random sampling 1000 different genome combinations of n genomes out of 67 genomes. Blue bars are the standard deviation of the medians. The red bars are weights used for model fitting and the red curve is the fitting result. **b)** Pan genome modeling of *S*. *mutans* genomes using three models, *y* = *a* + *bx*^*c*^, *y* = *a* − *bln*(*x* + *c*) and *y* = *a* × *e*^− *x*/*b*^ + *c* (where *a*, *b* and *c* are parameters), represented by green, blue and red curves respectively. Black rectangles are the medians of the pan-genome sizes calculated by random sampling 1000 different genomes combination of n genomes out of 67 genomes, and black bars are the standard deviations of the medians.

The core-genome size of the 67 *S*. *mutans* strains was calculated to be 1,373. Detailed information of the core genes are provided in the Additional file 2. To estimate the theoretical core-genome size achievable with an infinite number of *S*. *mutans* genomes, core-genome size medians corresponding to different genome numbers as shown in Figure 3a by the red rectangles were first calculated by random sampling 1,000 genome combinations of n genomes out of the 67 *S*. *mutans* genomes. Then, we applied the exponential regression core-genome model Fcn=κcexp−nτc+Ω proposed previously by Tettelin *et al*. [19,20] to fit the median data points of the core-genome sizes, where *K*_*c*_, τ_c_ and Ω are parameters, *n* represents the number of genomes, and Ω stands for the theoretical core-genome size. To take into consideration the different deviations of the core-genome size medians, as clearly indicated by the blue error bars in Figure 3a, we modified the fitting process by introducing the genome number as weight to the corresponding data point. The fitting parameters thus obtained are as follows: *r*^*2*^ = 0.97403, *K*_*c*_ = 325.74718 ± 10.00912,Ω = 1,369.41225 ± 1.986,τ_c_ = 15.90248 ± 0.66807 (Detailed information of all core and pan-genome modeling are given in Additional file 3). Using this fitting result to describe the core-genome of *S*. *mutans*, the theoretical core-genome size (Ω) was estimated to be around 1,370 genes, which is slightly lower than the calculated core-genome size (1,373) using 67 genomes. Compared with other streptococcus species, the core-genome of *S*. *mutans* is at the same level to the core-genome of *S*. *pyogenes* (1,400 genes determined using 11 strains), less than that of *S*. *pneumoniae* (1,647 genes determined using 47 *strains*) and *S*. *agalactiae* (1,800 genes determined using eight strains) [19,22,23]. However, we should be cautious with such comparison. In a recent study of Cornejo *et al*. [10], the core genome size of *S*. *mutans* was determined as 1,490 by using 57 *S*. *mutans* genomes which is obviously different to the core genome size of *S*. *mutans* we estimated, although we included the 57 *S*. *mutans* genomes used by Cornejo *et al*. in our study. The difference can be caused by different reasons, such as difference in the correction step for core gene determination and, very likely, different methods and parameter settings used for determining orthologs. Apparently, we have used a more stringent process to determine orthologs which led to smaller core genome size of *S*. *mutans* estimated.

### Pan-genome

We applied three models, namely *y* = *a* + *bx*^*c*^, y = a − b*l*n(*x* + *c*) and *y* = *a* × *e*^− *x*/*b*^ + *c* (where *a*, *b* and *c* are parameters) for modeling the pan-genome of *S*. *mutans*, as shown in Figure 3b by green, blue and red curves respectively (all fitting results are detailed in Additional file 3). Both the fitting results of using *y* = *a* + *bx*^*c*^ and y = a − b*ln*(*x* + *c*) indicated an infinite pan-genome, while the fitting result of using *y* = *a* × *e*^− *x*/*b*^ + *c* resulted in a negative value of the parameter *a*, suggesting a finite pan-genome However, the last fitting shows obvious deviations to many of the data points. Especially, the deviations even become larger with increased genome numbers, indicating that this model is not suitable. The best fitting result obtained with the model *y* = *a* + *bx*^*c*^ shows fittings to all the data points with very high confidence. Based on this model, the pan-genome of *S*. *mutans* is still “open” while 67 genomes were included, and the expected average new gene number with the addition of a new genome is estimated to be 15. The infinite pan-genome was first proposed by Tettelin *et al*. for *S*. *agalactiae* based on the use of 9 *S*. *agalactiae* genomes. The three regression models used in this study are all based on the assumption that contingency genes are independently sampled from the pan-genome with equal probability, except in the case of “specific/unique genes”, which are modeled as unique events that appear only once in the entire global population. Hogg *et al*. [24] proposed a finite supragenome model for pan-genome based on a different supposition that contingency genes are sampled from the pan-genome with unequal probability. By applying this finite supragenome model to 44 *S*. *pneumoniae* genomes, the predicted number of new genes drops sharply to zero when the number of genomes exceeds 50. However, in the case of *S*. *mutans* we could not observe such sharp decrease of new gene number even after 67 genomes were included. In the study of Cornejo *et al*. [10], they proposed a finite pan-genome for *S*. *mutans*, after they used a special “pseudogene cluster” identification process to exclude about 30% of the rare genes that are considered to be pseudogenes. However, they didn’t provide detailed parameters they obtained from fitting. Our modeling using the 67 *S*. *mutans* genomes by applying the model described above without any restrictions pointed to an infinite pan-genome of *S*. *mutans*. However, we would like to understand this predicted “infinite” pan-genome as follows: 1) a “pan-genome” should be considered as “dynamic” rather than “static”, which means the pan-genome content is changing during the evolution, it does not matter if its size is infinite or finite; 2) The change of a pan-genome content can be caused by the acquirement of new genes or by the loss of genes; 3) The actual pan-genome size can be more stable than the content of the pan-genome but can also change during evolution coupled with the change of the environment. Thus, without considering the “gene loss events”, it’s quite understandable to have a “growing” or “infinite” pan-genome as gene acquirement occurs no matter how slow it might be. Interestingly, Cornejo *et al*. found a high rate of LGT in *S*. *mutans*, where many genes were acquired from related streptococci and bacterial strains predominantly residing not only in the oral cavity, but also in the respiratory tract, the digestive tract, cattle, genitalia, in insect pathogens and in the environment in general [10]. Such high rate of LGT might also lead to a continuously growing pan-genome.

### Gene content-based comparative analysis of 10 mutans streptococci strains

The annotated protein sequences of all the genomes studied were cross-compared based on alleles/ortholog groups established by the program OrthoMCL [25]. In total, 2,211 putative alleles/ortholog groups are established, as documented in Additional file 4. A pair-wise comparison of the protein coding sequences between each two strains is shown in Table 2. It is clear to see that remarkable differences in protein coding sequences exist between the strains, even inside the same species of *S*. *mutans*. In the following sections, systems that are highly related to stress resistance and pathogenicity are presented and discussed. As all the following results are based on putative alleles/ortholog groups established by OrthoMCL, if not otherwise specified, the word “putative allele(s)/ortholog(s)” is omitted in the following text.

**Table 2:** **Unique protein coding sequences** (**CDSs**) **revealed by ortholog analysis between the different strains of this study**

	**Unique CDSs in comparision to**										
	***S. mutans *UA159**	***S. mutans *NN2025**	***S. mutans *5DC8**	***S. mutans *KK21**	***S. mutans *KK23**	***S. mutans *AC4446**	***S. mutans *ATCC 25175**	***S. mutans *NCTC 11060**	***S. ratti *DSM 20564**	***S. sobrinus *DSM 20742**	**All others**
***S***. ***mutans *UA159**		216	125	63	230	221	166	212	427	566	42
***S***. ***mutans *NN2025**	150		150	150	133	102	182	167	358	510	24
***S***. ***mutans *5DC8**	85	176		52	164	161	132	153	379	522	31
***S***. ***mutans *KK21**	47	200	76		190	184	127	175	402	544	3
***S***. ***mutans *KK23**	183	152	157	159		146	173	175	387	525	56
***S***. ***mutans *AC4446**	145	92	125	124	117		159	146	364	502	31
***S***. ***mutans *ATCC 25175**	117	199	123	94	171	186		146	373	525	33
***S***. ***mutans *NCTC 11060**	126	147	107	105	136	136	109		334	488	34
***S***. ***ratti *DSM 20564**	432	429	424	423	439	445	427	425		564	289
***S***. ***sobrinus *DSM 20742**	616	626	612	610	622	628	624	624	609		492

### High diversities of the competence development regulation module

In a previous study we have systematically discussed the two-component signal transduction systems (TCSTSs) in the 10 mutans streptococci strains [26]. ComDE, one of the TCSTS is directly related to competence development. Competence development is a complex process involving sophisticated regulatory networks that trigger the capacity of bacterial cells to take up exogenous DNA from the environment. This phenomenon is frequently encountered in bacteria of the oral cavity, e.g., *S*. *mutans*[27]. In *S*. *mutans*, ComX, an alternative sigma factor, drives the transcription of the so called ‘late-competence genes’ required for genetic transformation. ComX activity is modulated by the inputs from two types of signal pathways, namely the competence-stimulating peptide (CSP) dependent competence regulation system and CSP-independent competence regulation system. ComX and the ‘late-competence genes’ regulated by ComX as labeled by boldface in Table 3, are highly conserved even between the species, indicating that all mutans streptococci studied here might have the potential ability of transforming to genetic competence state. On the other hand, the upstream signal pathways regulating the activity of ComX show high variety as discussed in details below.

**Table 3:** **Distribution of competence development**-**related systems in the 10 mutans streptococci strains**

**Name**	**Function**	***S. mutans***	***S. mutans***	***S. mutans***	***S. mutans***	***S. mutans***	***S. mutans***	***S. mutans***	***S. mutans***	***S. ratti***	***S. sobrinus***
		**UA159**	**NN2025**	**5DC8**	**KK21**	**KK23**	**AC4446**	**ATCC 25175**	**NCTC 11060**	**DSM 20564**	**DSM 20742**
ComA/	Competence factor & nonlantibiotic mutacin transporting ATP-binding/permease protein	*SMU.286*	*GI|290581206*	*D816_01150*	*D817_01300*	*D818_01134*	*D819_01163*	*D820_01336*	*D821_01208*	*D822_01584*	*D823_05343*
		SMU.1881c	GI|290579788	D816_08453	D817_08643	-	D819_07724		D821_08449	D822_08325-	D823_01400
NlmT											
ComB/	Accessory factor for NlmT	SMU.287	GI|290581205	D816_01155	D817_01305	D818_01139	D819_01168	D820_01341	D821_01213	D822_01589	D823_05923
NlmE											
ComC	*S. mutans* specific competence stimulating peptide, precursor	SMU.1915	GI|290579762	D816_08588	D817_08778	D818_08368	D819_07839	D820_08520	D821_08549	-	-
SepM	cell surface-associated protease; Cleavage CSP.	SMU.518	GI|290580977	D816_02205	D817_02448	D818_02735	D819_02254	D820_02420	D821_02274	D822_04126	D823_08607
ComD	histidine kinase	SMU.1916	GI|290579761	D816_08593	D817_08783	D818_08373	D819_07844	D820_08525	D821_08554		D823_05333
ComE	response regulator	SMU.1917	GI|290579760	D816_08598	D817_08788	D818_08378	D819_07849	D820_08530	D821_08559		*D823_05328*
											D823_7992
HtrA	serine protease	SMU.2164	GI|290581420	D816_09733	D817_00015 D817_09913	D818_00020	D819_09056	D820_09650	D821_09748	D822_05851	D823_03191
HdrM	high density responsive membrane protein	SMU.1855	GI|290579809	D816_08353	D817_08543	D818_08143	D819_07614	D820_08345	D821_08319	D822_08240	D823_08222
HdrR	high density responsive regulator	SMU.1854	GI|290579810	D816_08348	D817_08538	D818_08138	D819_07609	D820_08340	D821_08314	-	-
BrsM		SMU.2081	GI|290581347	D816_09358	D817_09538	D818_09198	D819_08671	D820_09275	D821_09348	-	-
BrsR		SMU.2080	GI|290581346	D816_09353	D817_09533	D818_09193	D819_08666	D820_09270	D821_09343	D822_05085	-
OppD	oligopeptide ABC transporter	SMU.258	GI|290581226	D816_01025	D817_01175	D818_01039	D819_01063	D820_01211	D821_01051	D822_05611	D823_04322
ComS	*comX*-inducing peptide (XIP) precursor	NC_004350.2	NC_013928.1	D816_00277	D817_00297	D818_00297	D819_00203	D820_00247	D821_00253	D822_01077	-
		(62613 - 62666)a	(60952- 61005)b								
ComR	ComS receptor	SMU.61	GI|290579576	D816_00275	D817_00295	D818_00294	D819_00200	D820_00245	D821_00250	D822_01080	
ComX(SigX)	competence-specific sigma factor	SMU.1997	GI|290579687	D816_08973	D817_09163	D818_08748	D819_08219	D820_08900	D821_08929	D822_07328	D823_08887
**ComEA**	**competence protein**	**SMU.625**	**GI|290580890**	**D816_02675**	**D817_02923**	**D818_03217**	**D819_02694**	**D820_02880**	**D821_02784**	**D822_02674**	**D823_08107**
**ComEC**	**competence protein; possible integral membrane protein**	**SMU.626**	**GI|290580889**	**D816_02680**	**D817_02928**	**D818_03222**	**D819_02699**	**D820_02885**	**D821_02789**	**D822_02679**	**D823_08117**
**CoiA**	**competence protein CoiA**	**SMU.644**	**GI|290580870**	**D816_02775**	**D817_03018**	**D818_03322**	**D819_02786**	**D820_02970**	**D821_02879**	**D822_02739**	**D823_01025**
**EndA**	**competence associated membrane nuclease (DNA-entry nuclease)**	**SMU.1523**	**GI|290580108**	**D816_06842**	**D817_07008**	**D818_06659**	**D819_06647**	**D820_06860**	**D821_06857**	**D822_03254**	**D823_09687**
**ComG**	**competence protein G**	**SMU.1981c**	**GI|290579702**	**D816_08898**	**D817_09088**	**D818_08673**	**D819_08144**	**D820_08825**	**D821_08854**	**D822_07418**	**D823_01170**
**ComYD**	**competence protein ComYD**	**SMU.1983**	**GI|290579700**	**D816_08908**	**D817_09098**	**D818_08683**	**D819_08154**	**D820_08835**	**D821_08864**	**D822_07408**	**D823_01160**
**ComYC**	**competence protein ComYC**	**SMU.1984**	**GI|290579699**	**D816_08913**	**D817_09103**	**D818_08688**	**D819_08159**	**D820_08840**	**D821_08869**	**D822_07403**	**D823_01155**
	**possible competence-induced protein**	**SMU.2075c**	**GI|290581342**	**D816_09328**	**D817_09508**	**D818_09168**	**D819_08641**	**D820_09245**	**D821_09318**	**D822_05110**	**D823_03558**
**CinA**	**competence damage-inducible protein A**	**SMU.2086**	**GI|290581351**	**D816_09383**	**D817_09563**	**D818_09218**	**D819_08691**	**D820_09295**	**D821_09368**	**D822_05060**	**D823_03593**
**ComYB**	**competence protein; general (type II) secretory pathway protein**	**SMU.1985**	**GI|290579698**	**D816_08918**	**D817_09108**	**D818_08693**	**D819_08164**	**D820_08845**	**D821_08874**	**D822_07398**	**D823_01150**
**ComYA**	**late competence protein; type II secretion system protein**	**SMU.1987**	**GI|290579697**	**D816_08923**	**D817_09113**	**D818_08698**	**D819_08169**	**D820_08850**	**D821_08879**	**D822_07393**	**D823_01145**
**ComFC**	**late competence protein required for DNA uptake**	**SMU.499**	**GI|290580999**	**D816_02100**	**D817_02348**	**D818_02650**	**D819_02154**	**D820_02290**	**D821_02159**	**D822_06218**	**D823_02981**
**ComFA**	**late competence protein F**	**SMU.498**	**GI|290581000**	**D816_02095**	**D817_02343**	**D818_02645**	**D819_02149**	**D820_02285**	**D821_02154**	**D822_06223**	**D823_02986**
**CinA**	**competence damage-inducible protein A;**	**SMU.2086**	**GI|290581351**	**D816_09383**	**D817_09563**	**D818_09218**	**D819_08691**	**D820_09295**	**D821_09368**	**D822_05060**	**D823_03593**

In the case that more than one paralogs were found, the most similar ortholog is marked in italic. The rows related to highly conserved ‘late-competence genes’ were shown in boldface. The missing of ComDE in *S*. *ratti* DSM 20564 has been published in our previous study [26].

^*a*^location of *comS* in *S*. *mutans* UA159.

^b^location of *comS* in *S*. *mutans* NN2025.

#### CSP-dependent competence regulation system

It has been reported that the ComABCDE system in *S*. *mutans* combines the action of the two ortholog systems which are present as ComABCDE and BlpABCRH in *S*. *pneumoniae* and involved in competence regulation and bacteriocins regulation, respectively. It should be noticed that, ComAB have been primarily considered to be the transporter of ComC, the precursor of CSP. Later, ComAB have been renamed as NlmTE as they were found to function together as transporter of nonlantibiotic bacteriocins, while another gene pair CslAB was supposed to be the transporter of ComC [28]. However, a recent study confirms that ComAB is indeed a transporter both for nonlantibiotic bacteriocin and the peptide pheromone CSP [29].

**Figure 4:**
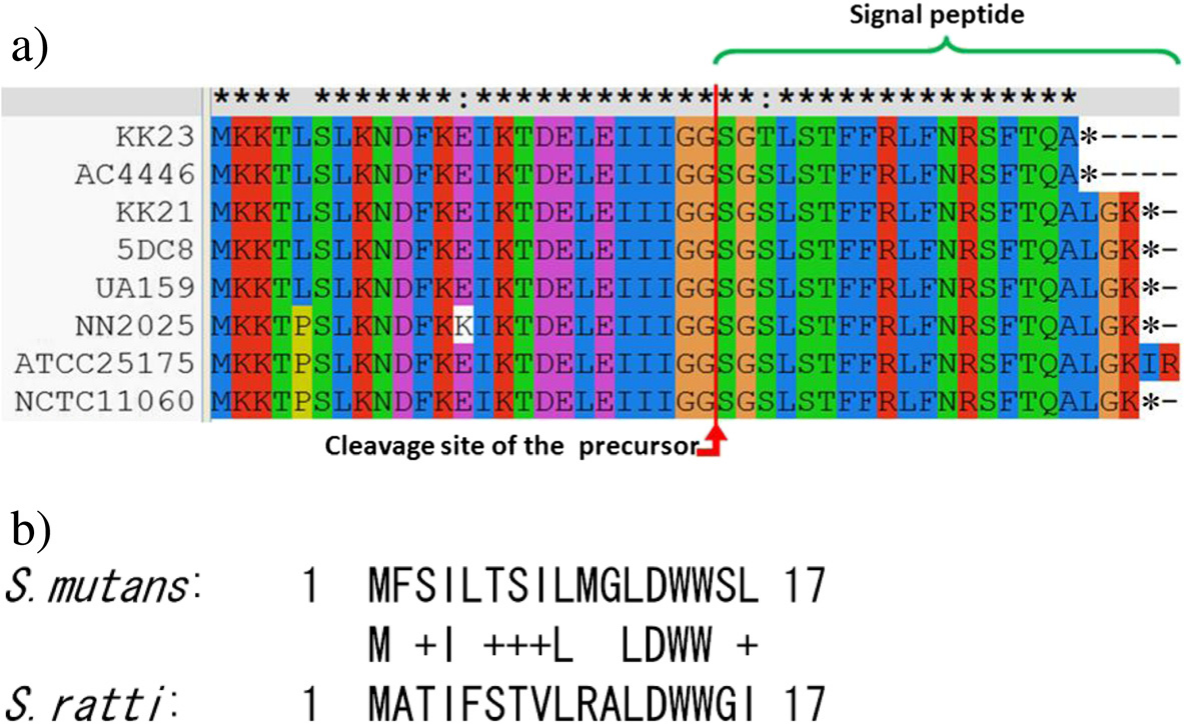
**Alignment of ComC and ComS amino acid sequences. a)** Alignment of ComC amino acid sequences identified in *S*. *mutans* species using CLUSTALX. Conserved residues are marked with “*” above the figure. The diversity in the ComC sequences have been verified by PCR experiments (data not shown). **b)** BlastP alignment of the ComS sequence of *S*. *mutans* (identical among the eight *S*. *mutans* strains) with that of *S*. *ratti* DSM 20564 (No ComS was identified in *S*. *sobrinus*). “+” stands for similar amino acid residues.

In *S*. *mutans*, the *comC*-encoded prepeptide of CSP has a leader sequence containing a conserved double glycine (GG), at which the leader sequence is cleaved during transporting by ComAB to generate the mature signal peptide (CSP-21) containing 21 amino acid residues [28,30,31]. Recent studies show that an extracellular protease, SepM (SMU.518), is involved in the further processing of CSP-21 by removing the “LGK” residues in the C-terminal to generate a 18-residue peptide (CSP-18), which can work at a concentration much lower than that of CSP-21 [29,32]. SepM is identified in all the 10 strains compared in this study, although putative *comC* alleles are present only in the eight *S*. *mutans* strains, not in the *S*. *sobrinus* DSM 20742 and *S*. *ratti* DSM 20564. Multi-alignment of the ComC sequences shows clear variations among different *S*. *mutans* strains (Figure 4a). Genetic variation of ComC in *S*. *mutans* has been reported previously [33]. Interestingly, the C-terminal amino acid sequence “LGK” of ComC is absent in the ComC prepetides of *S*. *mutans* KK23 and AC4446, which have also been observed previously in other *S*. *mutans* strains by Allan *et al*. [33]. ATCC 25175 possesses a unique ComC sequence ended with “LGKIR” at its C-terminal. In addition to the variations at the carboxyl end, substitutions of single amino acid residues at different positions are also found.

We have verified all the variants of *comC* revealed in this study by PCR experiments. Although Allan *et al*. pointed out that different *comC* alleles in some clinical strains of *S*. *mutans* exist but their products are functionally equivalent and there is no evidence of phenotype specificity [33], considering the complexity of phenotype evaluation, whether and how the variations found in this study may affect the natural genetic competence of these *S*. *mutans* strains requires further investigation.

The CSP-initiated activation of the response regulator ComE, through its cognate receptor kinase ComD, leads to the induction of competence through the alternative sigma factor ComX, and at the same time ComE directly induces a set of bacteriocin-related genes [28,30,34-38]. In our previous study focused on the comparison of the two-component signal transduction systems of these mutans streptococci strains, we have reported the complete missing of ComDE in *S*. *ratti* DSM 20564 and the low similarities of putative ComDE in *S*. *sobrinus* DSM 20742 to the ComDE of *S*. *mutans* strains [26]. Accordingly, no *comC*-like genes could be identified in *S*. *ratti* DSM 20564 and *S*. *sobrinus* DSM 20742. Thus, it can be inferred that *S*. *ratti* DSM 20564 and *S*. *sobrinus* DSM 20742 are totally different to the *S*. *mutans* strains regarding cellular functions including genetic competence associated with the ComABCDE system.

In *S*. *mutans*, no binding motif for ComE is present in the promoter region of ComX, suggesting that ComE is not a direct regulator of ComX, whereas a new peptide regulator system (ComSR) downstream of ComE that directly activates ComX has been identified by Mashburn-Warren *et al*. ComR activates the expression of the ComS, which is secreted, processed, and internalized through the peptide transporter OppD. The processed peptide, designated XIP (for sigma X-inducing peptide), modulates the activity of ComR, which in turn activates the expression of ComX. Deletion of *comR* or *comS* gene completely abolished the competence in *S*. *mutans*[39]. In this study, the ComSR regulating system is identified in most of the strains, except for *S*. *sobrinus* DSM 20742 which lacks the ComSR-coding genes. This well explains the fact that despite the presence of *comX* and the ‘late-competence genes’ we were not able to obtain the genetic competence state of *S*. *sobrinus* DSM 20742 (see discussion later in the “Variability and specificity in metabolic pathways and network” part). It is also worth to mention that the putative ComS ortholog found in *S*. *ratti* DSM 20564 is quite different to those of *S*. *mutans* strains, as shown in Figure 4b.

#### CSP-independent competence regulation system

It has been reported that a basal level of competence remains (referred as CSP-independent competence) after the deletion of *comE* from *S*. *mutans*, suggesting that the CSP-dependent regulation system is one of the several signaling pathways involved in ComX activation [34]. Indeed, under conditions of biofilm growth the HdrMR system, a novel two-gene regulatory system, has been shown to contribute to competence development through the activation of ComX by a yet unknown signal [40]. Moreover, microarray analysis revealed that both regulators, ComE and HdrR, activate a large set of overlapping genes [40,41]. Recently, Xie *et* al. identified in *S*. *mutans* another regulatory system, designated BsrRM, that primarily regulates bacteriocin-related genes but also affects the HdrMR system and thus indirectly contributes to competence development [42]. In this study, HdrR, the response regulator of the HdrMR system, is found neither present in *S*. *ratti* DSM 20564 nor in *S*. *sobrinus* DSM 20742. Furthermore, the response regulator BrsR of the BsrRM system is also absent in *S*. *ratti* DSM 20564, whereas *S*. *sobrinus* DSM 20742 lacks the complete BsrRM system. It’s also worth to mention that a competence damage-inducible protein CinA, which is regulated via ComX and has been proven to be related to DNA damage, genetic transformation and cell survival [43], is present in all strains.

Taking together, both the CSP-dependent and CSP-independent competence regulation systems in *S*. *ratti* DSM 20564 and especially in *S*. *sobrinus* DSM 20742 are very different to those of the *S*. *mutans* strains.

### Distribution of bacteriocin-related proteins and antibiotic resistance-related proteins

#### Bacteriocin-related proteins

Bacteriocins are proteinaceous toxins produced by bacteria to kill or inhibit the growth of similar or closely related bacterial strain(s). Bacteriocins produced by mutans streptococci are named “mutacins”. As dental plaque, the dominating niche of mutans streptococci, is a multispecies biofilm community that harbors many microorganism species, mutans group strains have developed a variety of mutacins to inhibit the growth of competitors, such as mitis group streptococci [44-46]. In this study, information about known mutacins as well as mutacin-immunity proteins was collected from the NCBI (http://www.ncbi.nlm.nih.gov) and Oralgen (http://www.oralgen.lanl.gov/) databases, as well as by searching for related publications. The collected protein sequences, as listed in Additional file 5, were used to blast against the proteomes of the 10 strains to see whether or not these known mutacins and mutacin-immunity proteins do exist in the mutans streptococci strains of this study. Distributions of identified mutacins and mutacin-immunity proteins are summarized in Table 4. Using this approach it is, however, not possible to identify any new types of mutacins.

**Table 4:** Distribution of mutacins and mutacin immunity proteins in the 10 mutans streptococci strains

	***S. mutans *UA159**	***S. mutans *NN2025**	***S. mutans *5DC8**	***S. mutans *KK21**	***S. mutans *KK23**	***S. mutans *AC4446**	***S. mutans *ATCC 25175**	***S. mutans *NCTC 11060**	***S. ratti***	***S. sobrinus *DSM 20742**	**References**
									**DSM 20564**		
**Mutacin**-**SMB** (**lantibiotic mutacin**)	-	-	-	-	-	-	-	-	D822_07608 D822_07613	-	[47,48]
**Mutacin**-**I** (**lantibiotic mutacin**)	-	-	-	-	-	-	-	-	-	-	[49,50]
**Mutacin**-**II** (**lantibiotic mutacin**)	-	-	-	-	-	-	-	-	-	-	[51]
**Mutacin**-**III** (**lantibiotic mutacin**)	-	-	-	-	-	-	-	-	-	-	[52]
**Mutacin**-**K8** (**lantibiotic mutacin**)	-	GI|290579849 GI|290579848 GI|290579850	-	-	D818_07928 D818_07933 D818_07938	-	-	-	-	-	[53]
**Mutacin**-**IV** (**NlmA**)	SMU.150	-	D816_00655	D817_00675	D818_00659	-	D820_00642	D821_00661	-	-	[54]
**Mutacin**-**IV** (**NlmB**)	SMU.151	-	D816_00660	D817_00680	D818_00664	-	D820_00647	D821_00666	-	-	[54]
**Mutacin**-**IV like** (**SMU**.**283**)	SMU.283	GI|290581209	D816_01135	D817_01285	D818_01099	D819_01148	D820_01321	D821_01193	D822_03404	-	[8]
**Immunity protein of Mutacin**-**IV**	SMU.152	GI|290580110	D816_06832	D817_06998	D818_06649	D819_06637	D820_06850	D821_06847	D822_03264	D823_04636	[55,56]
**Mutacin**-**V** (**CipB**)	SMU.1914c	GI|290579763	D816_08583	D817_08773	D818_08363	D819_07834	-	-	D822_03354	-	[55,56]
**CipI**, **immunity protein of CipB** (**Mutacin**-**V**)	SMU.925		D816_04020	D817_04283	D818_04522	D819_04119	D820_04232	D821_04089	D822_03349		[55,56]
**Homolog of CipI**	SMU.1913c	GI|290579764	D816_08578	D817_08768	D818_08358	D819_07829				D823_03992	[56]
**SMU**.**423 possible bacteriocin**	SMU.423	GI|290581063	D816_01775	D817_01930	D818_01847	D819_01823	D820_01975	D821_01862		D823_05348	[8]
**NlmT**/**ComA** transporting ATPase	**SMU**.**286** SMU.1881c	**GI**|**290581206** GI|290579788	**D816**_**01150** D816_08453	**D817**_**01300** D817_08643	**D818**_**01134** -	**D819**_**01163** D819_07724	**D820**_**01336** -	**D821**_**01208** D821_08449	**D822**_**01584** D822_08325 -	**D823**_**05343** D823_01400	[28,29]
**NlmE**/**ComB** accessory factor for NlmT	SMU.287	GI|290581205	D816_01155	D817_01305	D818_01139	D819_01168	D820_01341	D821_01213	D822_01589	D823_05923	[28,29]

In the case that more than one paralogs were found, the most similar ortholog is marked in bold font.

Note: as a multi-function exporter, the entries of NlmTE(ComAB) have been shown in Table 3 and here again.

**Figure 5:**
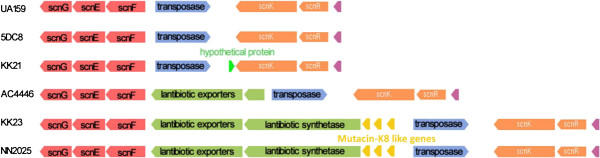
**Cluster structure of the mutacin**-**K8 production system across six *S. mutans *strains.** The ORFs colored in yellow are the possible mutacin-K8 precursor genes. *scnGEF*: bacteriocin related ABC element; possible immunity system; *scnK*: histidine kinase of two component system; *scnR*: response regulator of two component system (*Note*: *mutacin*-*K8 production system was failed to be identified in S*. *mutans* NCTC 11060, *S*. *mutans* ATCC 25175, *S*. *ratti* DSM 20564 *and S*. *sobrinus* DSM 20742).

Diversity of *Streptococcus* bacteriocins has been reported previously [57,58]. The mutacin assortments among the 10 strains in this study also demonstrate certain variations. An interesting result is that in contrast to *S*. *mutans* strains and *S*. *ratti* DSM 20564, *S*. *sobrinus* DSM 20742 does not possess any genes coding for mutacin-like proteins. Mutacin-SMB has been identified in *S*. *mutans* and *S*. *ratti* previously [47,48]. In our study, mutacin-SMB cluster was only identified in *S*. *ratti* DSM 20564 comprising 7 genes, including the mutacin-coding genes *smbA* and *smbB*, as well as 5 mutacin-related genes (*smbG*- > D822_07603, *smbT*- > D822_07593, *smbM*- > D822_07578, *smbF*- > D822_07588, and *smbM2*- > D822_07598). Lantibiotic mutacins, mutacin-I [49], mutacin-II [51] and mutacin-III [52], are completely absent in the 10 mutans streptococci strains. However, three gene copies possibly encoding the precursor of the lantibiotic mutancin mutacin-K8 are identified in the *S*. *mutans* strains KK23 and NN2025. Mutacin-K8 is an ortholog of the bacteriocin Streptococcin A-FF22 identified in group-A streptococci [59], and its production system has previously also been identified in the *S*. *mutans* strain K8 [53]. By carefully examining the genes surrounding mutacin-K8 precursor genes the gene cluster coding for a complete mutacin-K8 production system is also revealed in the strains KK23 and NN2025 (Figure 5). A partial ortholog of the mutacin-K8 production system is found in *S*. *mutans* UA159, 5DC8 and KK21, with only genes responsible for the immunity (*scnFEG*) left behind. Orthologous genes coding for a part of the mutacin-K8 production system are also found in *S*. *mutans* AC4446, consisting of only *scnFEG*, *scnT* (coding a lantibiotic exporter) and a part of *scnM* (coding the lantibiotic synthetase). Since a gene encoding ISSmu2-type transposase is found to be located upstream of mutacin-K8 precursor genes, we infer that the variety of mutacin-K8 production system in *S*. *mutans* strains studied here is highly possible to be caused by transposase actions.

Mutacin-IV, nonlantibiotic bacteriocins coded by *nlmA*/*B* (SMU.150/151, Note: hereinafter whenever needed/possible the locus_tag of the reference strain S. mutans UA159 is given for convenience) was discovered first in *S*. *mutans* UA140 to be active against the mitis group streptococci [54]. In this study, *nlmA*/*B* are found to be present in six of the *S*. *mutans* strains, including UA159, 5DC8, KK21, KK23, ATCC 25175 and NCTC 11060, but not in *S*. *mutans* NN2025 and AC4446, nor in *S*. *ratti* DSM 20564 and *S*. *sobrinus* DSM 20742. On the other hand, the immunity protein for mutacin-IV (SMU.152), is identified in all strains, consistent with the fact that no inhibition phenomenon has been observed yet among different mutans streptococci strains. A mutacin-IV like protein found before in the strain UA159 (SMU.283) is identified in all strains except for *S*. *sobrinus* DSM 20742.

Mutacin-V, another nonlantibiotic peptide coded by *cipB* (SMU.1914) is found, in addition to *S*. *sobrinus* DSM 20742, also absent in the *S*. *mutans* strains ATCC 15175 and NCTC 11060. There are two homologs of mutacin-V immunity protein in *S*. *mutans* UA159, namely CipI (SMU.925) and SMU.1913 [8,60]. These two immunity proteins share a sequence identity of 82%. However, it has been reported that though very likely co-transcribed with *cipB*, SMU.1913 cannot prevent CipB-caused cell lysis in *S*. *mutans* UA159, and the key immunity factor of mutacin-V has been supposed to be CipI (SMU.925) rather than SMU.1913 [60]. All the 10 strains including *S*. *sobrinus* DSM 20742 possess at least one orthologous gene encoding one of the two mutacin-V immunity proteins. Based on the similarity scores *S*. *mutans* NN2025 does not have an ortholog of CipI (SMU.925), but it possesses an ortholog (GI|290579764) of SMU.1913, which is possibly co-transcribed with GI|290579764, the *cipB* ortholog in *S*. *mutans* NN2025. Furthermore, the only putative immunity protein D822_3349 in *S*. *ratti* DSM 20564 shows very close similarities to SMU.925 (61%) and SMU.1913 (56%) and is possibly co-transcribed with D822_03354, the CipB ortholog in *S*. *ratti* DSM 20564. From these results, we suppose that SMU.1913, which is co-transcribed with *cipB* (SMU.1914), might be the ancestor gene coding for the mutacin-V immunity factor. The additional copy, like SMU.925 in *S*. *mutans* UA159, might be generated by duplication action and evolved as the dominant immunity factor in some of the mutans streptococci strains.

Furthermore, a possible nonlantibiotic bacteriocin peptide (SMU.423) is found to be present in all strains except for *S*. *ratti* DSM 20564. Putative ComAB, which has been proved to be the transporter complex of mutacin IV in *S*. *mutans*[28], are identified in all strains, supporting the suggestion that ComAB might function as a common transporter for multi-type nonlantibiotic bacteriocins rather than merely for mutacin IV. Moreover, an additional paralog of ComA is present in most of the strains except for *S*. *mutans* KK23 and *S*. *mutans* ATCC 25175.

To summarize, a differed distribution of mutacin/bacteriocin encoding genes accompanied with a high conservation of genes coding for mutacin-immunity proteins are revealed for the 10 mutans streptococci strains/species. The conservation of mutacin immunity proteins apparently plays an important role for the survival of mutans streptococci strains under a bacteriocin-rich environment.

#### Antibiotic resistance-related proteins

Bacteria and other microorganisms that cause infections are remarkably resilient and can develop ways to survive drugs meant to kill or weaken them. Antibiotic resistance can be a result of horizontal gene transfer [61], and also of unlinked point mutations in the pathogen genome at a rate of about 1 in 10^8^ per chromosomal replication [62]. The antibiotic action against the pathogen can be seen as an environmental selective pressure and bacteria which have developed mutations allowing them to survive will live on to reproduce. They will then pass this trait to their offsprings, which will result in the evolution of fully resistant colonies. Putative resistance-related genes are identified and listed in Table 5.

**Table 5:** **Distribution of antibiotic resistance**-**related proteins**

**Name**	**Putative function**	***S. mutans *UA159**	***S. mutans *NN2025**	***S. mutans *5DC8**	***S. mutans *KK21**	***S. mutans *KK23**	***S. mutans *AC4446**	***S. mutans *ATCC 25175**	***S. mutans *NCTC 11060**	***S. ratti *DSM 20564**	***S. sobrinus *DSM 20742**
**UppP**	Putative bacitracin resistant	SMU.244	GI|290581239	D816_00960	D817_01110	D818_00974	D819_00998	D820_01146	D821_00986	D822_05517	D823_09307
**BceA**	Bacitracin resistant ABC transporter ATP-binding protein	SMU.1006	GI|290580542	D816_04484	D817_04663	D818_04902	D819_04489	D820_04607	D821_04449	D822_02154	D823_04551
**BceB**	Bacitracin resistant ABC transporter permease protein	SMU.1007	GI|290580541	D816_04489	D817_04668	D818_04907	D819_04494	D820_04612	D821_04454	D822_02159	D823_04556
**DacF**	Penicillin binding protein; **Penicillin sensitive** protein	SMU.75	GI|290579588	D816_00335	D817_00355	D818_00354	D819_00260	D820_00330	D821_00310	D822_07803	D823_05036
**Pbp2X**	Penicillin-binding protein 2X; **Penicillin sensitive** protein	SMU.455	GI|290581039	D816_01905	D817_02153	D818_01967	D819_01954	D820_02095	D821_01964	D822_00802	D823_06528
	metallo-beta-Lactamase superfamily protein; **beta**-**Lactam resistance**;	SMU.368c	GI|290581108	D816_01525	D817_01680	D818_01583	D819_01608	D820_01711	D821_01583	D822_04346	D823_00655
	beta-Lactamase family protein; **beta**-**Lactam resistance**;	SMU.400	GI|290581086	D816_01660	D817_01815	D818_01732	D819_01708	D820_01860	D821_01747	D822_05706	D823_03675
**YqgA**	**beta**-**Lactam resistance**;	SMU.1444c	GI|290580186	D816_06482	D817_06653	D818_06314	D819_06285	D820_06483	D821_06502	D822_08877	D823_08387
	Lactamase_B;	SMU.1515	GI|290580115	D816_06807	D817_06973	D818_06624	D819_06612	D820_06825	D821_06822	D822_03289	D823_04661
	**beta**-**Lactam resistance**;										
**MurN**	**beta**-**Lactam resistance** factor MurN	SMU.716	GI|290580807	D816_03100	D817_03358	D818_03627	D819_03104	D820_03315	D821_03199	D822_00265	D823_09452
**MurM**	**beta**-**Lactam resistance** factor murM;	SMU.717	GI|290580806	D816_03105	D817_03363	D818_03632	D819_03109	D820_03320	D821_03204	D822_00260	D823_09457
	Macrolide-efflux protein		GI|290581182			D818_01269	D819_01313				
	Putative **multidrug resistance** ABC transporter		GI|290581181			D818_01274	D819_01318				
**VanW**	**Vancomycin b**-**type resistance** protein									D822_01634	
	Putative **multidrug resistance** protein b	SMU.745	GI|290580783	D816_03220	D817_03478	D818_03732	D819_03234 D819_09750	D820_03442	D821_03314	D822_00530	D823_08347
**PmrA**	Putative **multidrug resistance** efflux pump	SMU.1611c	GI|290580030	D816_07242	D817_07403	D818_07009	D819_07037	D820_07260	D821_07242	D822_07918	D823_02317
**YitG**	Putative **multidrug resistance**permease	SMU.1286c	GI|290580299	D816_05764	D817_05958	D818_02360	D819_05785	D820_05818	D821_05850	D822_01559	
	Putative **multidrug resistance**ABC transport	SMU.905	GI|290580642	D816_03940	D817_04208	D818_04447	D819_03949	D820_04157	D821_04009	D822_09885	D823_08492

The *S*. *mutans* species is known to be intrinsically resistant to bacitracins produced by *Bacillus subtilis*. We confirmed this by testing all the 10 strains with a bacitracin-E-test (data not shown). All strains including *S*. *ratti* DSM 20564 and *S*. *sobrinus* DSM 20742 had a minimum inhibitory concentration between 128 and >256 μg/l. In fact, this antibiotic is used to isolate mutans-streptococci from highly heterogeneous oral microflora. It has been reported that *bceABRS* (also named as *mbrABCD*) system, encoding a two component signal transduction system and an ABC-transporter, is required for bacitracin resistance in *S*. *mutans*[63,64]. As expected, ortholog of *bceABRS* system is found to be present in all strains. Furthermore, an ortholog of a putative bacitracin resistance protein UppP (SMU.244, undecaprenyl-diphosphatase) is present in all strains. It has been proved that overexpression of UppP in *Escherichia coli* and *Bacillus subtilis* results in bacitracin resistance [65,66]. However, the function of UppP in bacitracin resistance in mutans streptococci has not yet been investigated. Based on its conservation in all strains studied here, we suppose that UppP might play an important role in bacitracin resistance for mutans streptococci species as well.

Two penicillin-binding proteins (SMU.75 and SMU.455) are identified in all strains, indicating that they are potentially all susceptible to penicillin. Phenotypically all strains were tested to be susceptible to penicillin (data not shown). On the other hand, all the strains possess orthologs of SMU.368c, SMU.400, SMU.1444c and SMU.1515, which are homologs to beta-lactamases (EC 3.5.2.6), as well as orthologs of two so called beta-lactam resistance factors (SMU.716, SMU.717). Thus, all the strains are potentially capable of resistance against beta-lactam antibiotics. Orthologs of macrolide-efflux transporter proteins, as coded by GI|290581182 and GI|290581181 in *S*. *mutans* NN2025, are found to be also present in *S*. *mutans* 5DC8 and *S*. *mutans* KK21. A vancomycin b-type resistance-associated protein (D822_01634) is uniquely present in *S*. *ratti* DSM 20564, but our phenotypic testing showed as expected that *S*. *ratti* DSM 20564 is susceptible to vancomycin. Furthermore, several putative multidrug resistance-associated proteins (SMU.745, SMU.1611c and SMU.905 except for SMU.1286c) are found to be present in all strains.

### Oxidative stress defense systems in mutans streptococci

For protection against reactive oxygen species (such as O_2_^-^, H_2_O_2_, HO·) or adaptation to oxidative stresses aerobes and facultative anaerobes have evolved efficient defense systems, comprising an array of antioxidant enzymes such as catalase, superoxide dismutase (SOD), Dps-like peroxide resistance protein, alkylhydroperoxide reductase (AhpCF), glutathione reductase, and thiol reductase, which have been identified in many bacterial species.

Although the first genome sequence of *S*. *mutans* UA159 has already been published in 2002, the oxidative stress defense systems in the group of mutans streptococci have not yet been systematically discussed. By searching for known antioxidant systems in the genomes of the sequenced mutans streptococci strains of this study, we obtained an overview of putative oxidative defense systems in these mutans streptococci strains/species which are composed of superoxide dismutase (SOD), AhpF/AhpC system, Dpr, thioredoxin system and glutaredoxin system, as shown in Table 6.

**Table 6:** Distribution of oxidative stress resistance systems

**Class**	**Name**	**Function**	***S. mutans *UA159**	***S. mutans *NN2025**	***S. mutans *5DC8**	***S. mutans *KK21**	***S. mutans *KK23**	***S. mutans *AC4446**	***S. mutans *ATCC 25175**	***S. mutans *NCTC 11060**	***S. ratti *DSM 20564**	***S. sobrinus *DSM 20742**
**SOD**	Sod	Superoxide dismutase	SMU.629	GI|290580884	D816_02695	D817_02943	D818_03247	D819_02714	D820_02900	D821_02804	D822_02694	D823_08152
		3′-Phosphoadenosine-5′-phosphate phosphatase	SMU.1297	GI|290580288	D816_05819	D817_06013	D818_02305	D819_05840	D820_05873	D821_05905	D822_08440	D823_09052
**AhpF**/**AhpC system**	AhpC	Alkyl hydroper oxide reductase, subunit C	SMU.764	GI|290580768	D816_03290	D817_03548	D818_03807	D819_03314	D820_03512	D821_03389	D822_08028	-
	AhpF (Nox1)	Alkyl hydroperoxide reductase, subunit F	SMU.765	GI|290580767	D816_03295	D817_03553	D818_03812	D819_03319	D820_03517	D821_03394	D822_08023	-
**Dpr**	Dpr	Peroxide resistance protein / iron binding protein	SMU.540	GI|290580957	D816_02305	D817_02548	D818_02835	D819_02354	D820_02520	D821_02374	D822_04226	D823_02352
**Thioredoxin system**	TrxB	Thioredoxin reductase (NADPH)	SMU.463	GI|290581031	D816_01940	D817_02188	D818_02007	D819_01989	D820_02130	D821_01999	D822_06878	D823_01947
	TrxB	Thioredoxin reductase	SMU.869	GI|290580673	D816_03785	D817_04038	D818_04292	D819_03804	D820_04002	D821_03854	D822_03499	D823_01550
	TrxA	Thioredoxin	SMU.1869	GI|290579800	D816_08398	D817_08588	D818_08193	D819_07664	D820_08390	D821_08394	D822_08270	D823_06913
	TrxH	Thioredoxin family protein	SMU.1971c	GI|290579712	D816_08848	D817_09038	D818_08623	D819_08094	D820_08775	D821_08804	D822_07458	D823_08552
		Thioredoxin family protein	SMU.1169c	GI|290580401	D816_05229	D817_05413	D818_05692	D819_05219 D819_05259	D820_05307	D821_05309	D822_06958	-
	Tpx	Thiol peroxidase	SMU.924	GI|290580628	D816_04015	D817_04278	D818_04517	D819_04114	D820_04227	D821_04084	D822_03359	D823_07595
**Glutaredoxin system**	GshAB	Glutathione biosynthesis bifunctional protein	SMU.267c	GI|290581223	D816_01065	D817_01215	D818_01054	D819_01078	D820_01251	D821_01091	D822_01287	D823_06703
	GshR	Glutathione reductase	SMU.838	GI|290580702	D816_03640	D817_03893	D818_04147	D819_03659	D820_03857	D821_03709	D822_01904	D823_04976
	GshR	Glutathione reductase	SMU.140	-	D816_00620	D817_00640	D818_00624	-	D820_00607	D821_00626	D822_06143	-
	NrdH	Glutaredoxin	SMU.669c	GI|290580848	D816_02885	D817_03143	D818_03447	D819_02894	D820_03090	D821_03009	D822_02899	D823_05398

SOD, which catalyzes the dismutation of superoxide into oxygen and hydrogen peroxide, is an important antioxidant defense in nearly all cells exposed to oxygen [67]. SOD is found in all strains of this study. Catalase, which catalyzes the decomposition of hydrogen peroxide, is not found in any of the mutans streptococci strains of this study. It is known that although most streptococci can grow in the presence of air, they do not possess a catalase, implying that hydrogen peroxide defense mechanism, by which lactic acid bacteria established their growth in air, are very different to those of aerobes. It has been reported that both the bi-component peroxidase system AhpF/AhpC and Dps-like peroxide resistance protein confer tolerance to oxidative stress in *S*. *mutans*[68].

The AhpF/AhpC system catalyzes the NADH-dependent reduction of organic hydroperoxides and/or H_2_O_2_ to their respective alcohol and/or H_2_O. Both AhpF and AhpC are present in all *S*. *mutans* strains of this study and in *S*. *ratti* DSM 20564, but are absent in *S*. *sobrinus* DSM 20742. The natural missing of AhpF and AhpC in *S*. *sobrinus* indicates that AhpF/AhpC system is not an essential peroxide tolerance system for some mutans streptococci species. While studying a *ahpF* and *ahpC* double deletion mutant of *S*. *mutans*, Higuchi *et al*. [69] found that the mutant still showed the same level of peroxide tolerance as did the wild-type strain that led them to the finding of the *dpr* gene, which encodes a ferritin-like iron-binding protein involved in oxygen tolerance by limiting the nonenzymatic hydroxyl radical synthesis via iron-catalyzed ‘Fenton reaction’ in *S*. *mutans*. Their further studies on the biological function of *dpr* found that *dpr* gene from *S*. *mutans* chromosome was capable of complementing an alkyl hydroperoxide reductase-deficient mutant of *E*. *coli*, as well as complementing the defect in peroxidase activity caused by the deletion of *ahpF*/*ahpC* in *S*. *mutans*, indicating that *dpr* plays an indispensable role in oxygen tolerance of *S*. *mutans*[68,70]. Dpr homologs were found in all strains as expected by the supposed essential function of *dpr* gene in oxygen tolerance.

Thioredoxins are a class of small redox mediator proteins known to be present in all organisms. They are involved in many important biological processes, including redox signaling. Thioredoxins are kept in the reduced state by the flavor enzyme thioredoxin reductase in a NADPH-dependent reaction [71]. They act as electron donors to many proteins including thiol peroxidases [72]. Thioredoxin, thioredoxin reductase and thiol peroxidase, the components of thioredoxin system, are identified in all the strains of this study. Two putative thioredoxin reductases (SMU.463 and SMU.869) are found in all strains/species. It has been reported that in some species thioredoxin reductases have been evolved to be activated by both NADPH and NADH [73]. We speculate that SMU.463 and SMU.869 might have been evolved to have different preferences to NADPH and NADH (SMU.463 and SMU.869 shares less than 20% similarities). If it holds true, this could be advantageous for these mutans streptococci, as the extra amount of NADH produced from glycolysis/gluconeogenesis pathway under anaerobic conditions could be directly used for oxidative stress resistance. Thioredoxin (SMU.1869) and two thioredoxin family proteins (SMU.1971c and SMU.1169c) are found to be present in nearly all strains, except for *S*. *sobrinus* DSM 20742, which lacks any ortholog of SMU1169c. An ortholog of a thiol peroxidase-coding gene (*tpx*) is identified in all strains.

Glutaredoxins share many functions of thioredoxins but are reduced by glutathione (L-γ-glutamyl-L-cysteinylglycine, GSH) rather than by a specific reductase. This means that glutaredoxins are oxidized by their corresponding substrates, and reduced non-enzymatically by GSH [74]. Oxidized glutathione (GSSG) is then regenerated by glutathione reductase. Together, these components comprise the glutathione system [75]. GSH is a well-characterized antioxidant in eukaryotes and Gram-negative bacteria, where it is synthesized by the sequential action of two enzymes, γ-glutamylcysteine synthetase (γ-GCS) and glutathione synthetase (GS). Among Gram-positive bacteria only a few species contain GSH. It has been reported that streptococci lack the moderate-to-high levels of intracellular glutathione normally found in Gram-negative bacteria [76]. Using *Streptococcus agalactiae* as a model, it has been discovered that in GSH-containing Gram-positive bacteria GSH synthesis is catalyzed by one bifunctional protein, γ-glutamylcysteine synthetase-glutathione synthetase (γ-GCS-GS), encoded by one gene, *gshAB*. Homologs of γ-GCS-GS have been identified in the genomes of 19 mostly studied Gram-positive bacteria, including *S*. *mutans*[77]. All components of the glutathione system were identified in all the 10 strains of this study. Several *S*. *mutans* strains, namely UA159, 5DC8, KK21, KK23, ATCC 25175, and NCTC 11060, as well as *S*. *ratti* DSM 20564, possess two glutathione reductase orthologs (SMU.140 and SMU.838). This could possibly convey these strains certain advantages in the re-generation of GSH from GSSG, which in turn would be helpful for oxidative resistance.

In addition, 3′-phosphoadenosine-5′-phosphate phosphatase activity has recently been reported to be required for superoxide stress tolerance in *S*. *mutans*[78]. Putative 3′-phosphoadenosine-5′-phosphate phosphatase coding genes were identified in all strains (Table 6).

### Variability and specificity in metabolic pathways and network

In order to reveal the metabolic variability of the mutans streptococci systematically, we have reconstructed and analyzed the genome-scale metabolic networks of all the strains sequenced with the method proposed by Ma and Zeng [79] and an updated database [79]. All annotated protein sequences having EC numbers are considered for the network reconstruction. From the functional annotation discussed above, total EC numbers identified in the 10 strains are very close to each other, as shown in Table 7. A summary of the total numbers of the reactions and metabolites in each of the reconstructed metabolic networks is shown in Table 7, and all the constructed metabolic networks are provided in Additional file 6 in *.cys format which can be opened with Cytoscape [80], a software for visualization and analysis of biological networks. The sizes of the constructed metabolic networks of the eight *S*. *mutans* strains are very close to each other, with UA159, NN2025, AC4446, 5DC8 and KK21 having almost exactly the same size, and the networks of KK23, ATCC 25175 and NCTC 11060 being merely about 2% larger. While the size of the metabolic network of *S*. *ratti* DSM 20564 is comparable to those of the *S*. *mutans* strains, the metabolic network of *S*. *sobrinus* with 833 reactions and 853 metabolites is the smallest one, which have 62 less reactions and 60 less metabolites compared to the largest one of *S*. *mutans* NCTC 11060 (895 reactions and 913 metabolites).

**Table 7:** Compositions of the established metabolic networks of the 10 mutans streptococci strains

**Strain**	**EC numbers**	**Reactions**	**Metabolites**
***S***. ***mutans *UA159**	454	875	893
***S***. ***mutans *NN2025**	450	874	892
***S***. ***mutans *5DC8**	453	875	893
***S***. ***mutans *KK21**	453	875	893
***S***. ***mutans *KK23**	452	893	911
***S***. ***mutans *AC4446**	449	874	893
***S***. ***mutans *ATCC 25175**	453	891	911
***S***. ***mutans *NCTC 11060**	456	895	913
***S***. ***ratti *DSM 20564**	435	888	893
***S***. ***sobrinus *DSM 20742**	434	833	853

Despite the comparable network sizes, however, all the strains possess or lack certain reactions/metabolites, as revealed by detailed comparative analyses. Using the metabolic network of *S*. *mutans* UA159 as reference, the presence and absence of reactions in each of the strains/species compared are discovered and mapped into sub-pathways based on the KEGG pathway classification (http://www.genome.jp/kegg/pathway.html). As a result, among the 416 sub-pathways defined in the KEGG pathway database 46 sub-pathways demonstrated certain variations between the strains/species, as summarized in Additional file 7.

**Figure 6:**
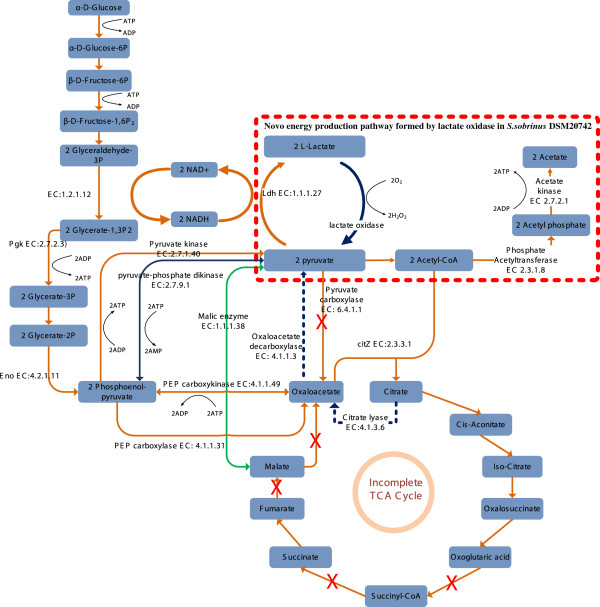
**Central metabolism pathways of mutans streptococci.** The orange lines represent enzyme reactions conserved across the mutans streptococci strains compared in this study, whereas the blue lines represent enzyme reactions specifically present (solid line) or absent (dashed line) in *S*. *sobrinus* DSM 20742. Reaction catalyzed by NAD^+^-specific malic enzyme (EC: 1.1.1.38) (green line) is absent in *S*. *mutans* NN2025 and *S*. *mutans* AC4446. Pyruvate-phosphate dikinase, catalyzing the interconversion of PEP and pyruvate (black line), is uniquely present in *S*. *ratti* DSM 20564.

A key feature of the oral environment is that the nutrients available to the oral bacteria are always fluctuating between abundance and famine associated with human diet. Thus, the ability to quickly acquire and metabolize carbohydrates to produce energy and precursors for biosynthesis is essential for the survival of all oral bacteria. Due to their key roles in carbohydrates metabolism and energy production, glycolysis/gluconeogenesis, TCA cycle and pyruvate metabolism pathways are generally considered to be highly conserved among these oral bacteria. Although mutans streptococci strains/species are closely related species as revealed by phylogenetic tree analysis in this study (Figure 1), differences in the central carbon metabolic pathways are found as shown in Figure 6.

Facultative anaerobes such as lactic acid bacteria including *Streptococcus* lack cytochrome oxidases required for energy-linked oxygen metabolism and energy (in the form of ATP) required for survival and growth are generated by substrate level phosphorylation in the glycolysis pathway [69]. L-lactate oxidase (D823_06598) with a similarity of 73% to YP_003064450.1 (accession number) of *Lactobacillus plantarum* JDM1 and lactate oxidase (D823_06595) with a similarity of 65% to ZP_09448656.1 (accession number) of *Lactobacillus mali* KCTC 3596, are found to be uniquely present in *S*. *sobrinus* DSM 20742. These two enzymes catalyze the reaction of L-Lactate + O_2_= > Pyruvate + H_2_O_2_ and/or D-Lactate + O_2_= > Pyruvate + H_2_O_2_. It has been reported that in *S*. *pneumoniae* concerted action of lactate oxidase and pyruvate oxidase forms a novel energy-generation pathway by converting lactate acid to acetic acid under aerobic growth conditions [81]. Because there is no pyruvate oxidase identified in *S*. *sobrinus* DSM 20742, the function of the lactate oxidases in *S*. *sobrinus* DSM 20742 should be different to that of *S*. *pneumoniae*. By a close examination we hypothesize that lactate oxidase, together with pyruvate dehydrogenase, phosphate acetyl transferase and acetate kinase, could form a novel energy production pathway to convert lactate acid to acetate and simultaneously produce one additional ATP, as depicted Figure 6. By doing so, the lactate oxidases of *S*. *sobrinus* DSM 20742 could also play a role in consuming lactate to regulate pH, which would be an advantage for *S*. *sobrinus* DSM 20742 in resistance to acid stress. In addition, this pathway could replenish Acetyl-CoA, an important intermediate for the biosynthesis of fatty acids and amino acids. This is for the first time that such an energy production pathway is proposed in *Streptococcus* species. Furthermore, lactate oxidase and lactate dehydrogenase could form a local NAD^+^ regeneration system, which would be certainly advantageous to *S*. *sobrinus* DSM 20742 under aerobic growth conditions. Moreover, it is known that mutans group streptococci and the mitis group streptococci are competitors, with *S*. *mutans* producing mutacins to kill the mitis group streptococci and the mitis group streptococci in turn produce H_2_O_2_ to kill mutans group streptococci [16,82]. Favored by possessing the lactate oxidases, *S*. *sobrinus* DSM 20742 has the potential ability of producing H_2_O_2_ to kill not only competitors (oxygen sensitive *S*. *mutans*, oral anaerobes) but also macrophages [83], and defend its ecological niche. The unique presence of lactate oxidases in *S*. *sobrinus* DSM 20742 was verified by PCR experiments as shown in Additional file 8. Later, we also found that another *S*. *sobrinus* strain AC153 also harbors homologous genes of lactate oxidase, suggesting that lactate oxidase may be conserved and play an important role in *S*. *sobrinus*. In the effort to clarify the functionality of lactate oxidase we tried to knock out the two genes encoding the two enzymes by PCR ligation mutagenesis according to the method of Lau PC *et al*. (2002). We applied different transformation methods (two natural transformation methods and two electroporation methods) but were failed to obtain the desired recombinants. Then, to find out if *S*. *sobrinus* DSM 20742 is able to enter genetic competence state at all, we tried to transform *S*. *sobrinus* with plasmids replicative in other Streptococcus spp. like pDL278 (Sp^r^, pAT18 Em^r^, with suicide vector pFW5 Sp^r^ in both circular and linearized forms but could not obtain the transformants. Therefore, it is clear that the genetic competence behavior of *S*. *sobrinus* DSM 20742 is very different to that of *S*. *mutans*, attributing very likely to the lacking of the genes comSR and *comC*.

In contrast to the unique harboring of lactate oxidases in *S*. *sobrinus* DSM 20742, citrate lyase (EC 4.1.3.6), which catalyzes the cleavage of citrate into oxaloacetate and acetate, and oxaloacetate decarboxylase (EC 4.1.1.3), catalyzing the irreversible decarboxylation of oxaloacetate to pyruvate and CO_2_, are not found in *S*. *sobrinus* DSM 20742, as shown in Figure 6 by the blue dotted lines. It has been reported that citrate lyase functions as a key enzyme in initiating the anaerobic utilization of citrate by a number of bacteria, further catabolism of oxaloacetate formed taking place either by decarboxylation or by reduction. In some organisms, oxaloacetate is decarboxylated to pyruvate by oxaloacetate decarboxylase, which is also induced in the presence of citrate. The two enzymatic reactions, which occur sequentially, constitute the ‘citrate fermentation pathway’ [84]. The absence of citrate lyase and oxaloacetate decarboxylase implies that *S*. *sobrinus* DSM 20742 might lacks the ability in anaerobic utilization of citrate as a substrate. The disadvantages of *S*. *sobrinus* DSM 20742 in citrate utilization could be offset by the novel energy production pathway from lactate to acetate proposed above.

A putative pyruvate-phosphate dikinase (EC 2.7.9.1), which catalyzes the interconversion between PEP and pyruvate, is found to be uniquely present in *S*. *ratti* DSM 20564. Pyruvate-phosphate dikinase has been found in propionic acid bacteria [85]. The large difference in the standard free energy of hydrolysis for ATP to AMP and pyrophosphate (−7.6 kcal/mole) and for PEP to pyruvate (−13.6 kcal/mole) at pH 7.0 indicates that the equilibrium for the reaction it catalyzes would strongly favor pyruvate formation. But studies in *Acetobacter xylinum* clearly indicate that the function of this enzyme under physiological conditions favors the process of gluconeogenesis [86]. Metabolite interconversion at the PEP-pyruvate-oxaloacetate node involves a structurally entangled set of reactions that interconnect the major pathways of carbon metabolism and thus, is responsible for the distribution of the carbon flux among catabolism, anabolism and energy supply of the cell [87]. Under glycolytic conditions oxaloacetate is generated by carboxylation of PEP and/or pyruvate catalyzed by PEP carboxylase (PEPCx) and/or pyruvate carboxylase (PCx). In this study PCx is not found in any of the mutans streptococci strains.

All the 10 strains of this study possess similarly an incomplete TCA cycle and the primary role of the existing TCA enzymes is most likely the synthesis of amino acid precursors as has been reported previously [8,88].

## Conclusion

In the present study, the genomes of 8 mutans streptococci strains, including six *S*. *mutans* strains, one *S*. *ratti* strain and one *S*. *sobrinus* strain were sequenced, annotated and compared together with *S*. *mutans* UA159 and NN2025. Multiple genome alignment showed extensive genome rearrangement among the eight strains of *S*. *mutans*. The core-genome size of *S*. *mutans* was determined to be around 1,370 genes by including 67 *S*. *mutans* genomes. A possibly open pan-genome of *S*. *mutans* was inferred.

Systematic comparative analyses were focused on competence regulation, bacteriocin (mutacin) production, antibiotic resistance, oxidative stress resistance, as well as central carbon metabolism and energy production pathways. Most of these systems show remarkable differences between the strains, except for oxidative stress resistance systems which are found to be well conserved. CSP-dependent and independent competence regulation systems are highly diverse in mutans streptococci: no *comC*-like genes could be identified in *S*. *ratti* and *S*. *sobrinus*; putative ComC amino acid sequences of *S*. *mutans* show clear variations; ComS and ComR are absent in *S*. *sobrinus* which well explains the fact that we were not able obtain genetic competence state of *S*. *sobrinus* by experiment, even though the ComX and the downstream competence development genes are well reserved; furthermore, the response regulators of the HdrMR and BsrRM systems, which are known to be involved in competence development, are missing in both *S*. *ratti* and *S*. *sobrinus*.

Variation in mutacin-encoding genes is accompanied with the conservation of mutacin immunity proteins, which indicates apparently important roles of the mutacin immunity proteins for the survival of these mutans streptococci in a bacteriocin rich environment. The presence of various antibiotic resistance factors, together with the open pan-genome inferred, implies that attention should be paid to the potential of mutans streptococci in the development of antibiotic resistance.

The sizes of the genome-scale metabolic networks of the 10 strains are very close to each other. Comparative analysis of sub-pathways reveals that 46 sub-pathways of all 416 sub-pathways defined in KEGG pathway database show variation using *S*. *mutans* UA159 as reference. By identifying lactate oxidases to be uniquely present in *S*. *sobrinus* DSM 20742, we proposed for the first time a novel energy production pathway in *S*. *sobrinus*. Additional functions of the lactate oxidases in connection with the proposed energy production pathway are also discussed.

In conclusion, the genomes of mutans streptococci display remarkable differences, especially among different species. We believe that the strain-specific information provided in this study should be helpful to understand the evolution and adaptive mechanisms of those oral pathogens.

## Methods

### Genome sequences and strains

All the newly sequenced strains were described previously [26]. Briefly, serotype c strain *S*. *mutans* 5DC8 was isolated from root caries by David Beighton (London, UK); serotype c strain *S*. *mutans* AC4446 was isolated from a proven case of infective endocarditis in Dillingen (Germany), serotype c strain *S*. *mutans* KK21 was isolated from enamel caries of an adult by Susanne Kneist (Jena, Germany), serotype c strain *S*. *mutans* KK23 was isolated from enamel caries of a child by Susanne Kneist (Jena, Germany), Serotype c, type strain *S*. *mutans* ATCC 25175 was isolated from carious dentine, serotype f strain *S*. *mutans* NCTC 11060 was isolated in Denmark from a patient’s blood, serotype b strain *S*. *ratti* DSM 20564(=ATCC 19645) was isolated from caries lesion in rat, and finally, serotype non-d & non-g strain *S*. *sobrinus* DSM 20742 (= ATCC 33478) was isolated from human dental plaque. Serotype c is over-represented because 70-80% of all *S*. *mutans* isolates are of this serotype. However, non-c serotypes seem to be associated with cardiovascular diseases and this is represented in our study by the serotype f strain. Besides *S*. *mutans*, *S*. *sobrinus* is considered as a relevant cariogenic species in human. The genome sequences of *S*. *mutans* UA159 and *S*. *mutans* NN2025 were genome sequenced previously and obtained from NCBI genome database (http://www.ncbi.nlm.nih.gov/genome/, consulted at the beginning of January 2011).

### Genome sequencing, assembly and annotation

All of the strains were sequenced using the Solexa sequencing platform at the Helmholtz Center for Infection Research in Braunschweig, Germany. The “high-quality draft” [89] genome sequences of these mutans streptococci strains were assembled by a combined use of the sequence assembly tools SOAPdenovo [90], Maq [91] and Phrap [92]. In brief, we first use SOAPdenovo to obtain the optimal assembly result by using different k-mer from 17 to 41 without scaffolding. Then we map all reads to reference sequence of *S*. *mutans* UA159 using Maq and break down the low quality area to obtain a collection of long contigs. Finally, the long contigs were used to close partial gaps of the initial assembly to improve the assembly quality using Phrap. The first version genome annotations were performed using mauve [13,93], tRNAscan-SE 1.21, Glimmer3.02 [94] and Blast2GO [95], and then released through our central genome database (http://biosystem.bt1.tu-harburg.de/) established with PathwayTools [26,96]. This version was used before for the study of TCSTSs of the 10 strains [26]. During this study, all genomes were re-annotated using the NCBI Prokaryotic Genomes Automatic Annotation Pipeline (PGAAP, http://www.ncbi.nlm.nih.gov/genomes/static/Pipeline.html) and the whole-genome shotgun sequences have been deposited at DDBJ/EMBL/GenBank under the accessions of AOBX00000000 (*S*. *mutans* 5DC8), AOBY00000000 (*S*. *mutans* KK21), AOBZ00000000 (*S*. *mutans* KK23), AOCA00000000 (*S*. *mutans* AC4446), AOCB00000000 (*S*. *mutans* ATCC 25175), AOCC00000000 (*S*. *mutans* NCTC 11060), AOCD00000000 (*S*. *ratti* DSM 20564) and AOCE00000000 (*S*. *sobrinus* DSM 20742). The present study used the new version deposited at DDBJ/EMBL/GenBank. As we found out that in the annotated results from PGAAP some coding genes are missing, we did manual curation based on blast searches using known coding nucleotide sequences, the location of the missing coding sequences are given in Additional file 9.

### Genome alignment

Multiple genome alignments were computed by using the progressive Mauve algorithm of the Mauve software [13] with default options.

### Core-genome and pan-genome analysis

In addition to the 6 *S*. *mutans* draft genomes of this study and the previously released complete genomes of *S*. *mutans* UA159 and NN2025, 59 newly released *S*. *mutans* genomes (2 completed and 57 drafts) available in NCBI till April 2013 were also included in the core- and pan-genome analysis of *S*. *mutans*. The accessions of the 59 genomes are as follows:

AGWE00000000, AHRB00000000, AHRC00000000, AHRD00000000, AHRE00000000, AHRF00000000, AHRG00000000, AHRH00000000, AHRI00000000, AHRJ00000000, AHRK00000000, AHRL00000000, AHRM00000000, AHRN00000000, AHRO00000000, AHRP00000000, AHRQ00000000, AHRR00000000, AHRS00000000, AHRT00000000, AHRU00000000, AHRV00000000, AHRW00000000, AHRX00000000, AHRY00000000, AHRZ00000000, AHSA00000000, AHSB00000000, AHSC00000000, AHSD00000000, AHSE00000000, AHSF00000000, AHSG00000000, AHSH00000000, AHSI00000000, AHSJ00000000, AHSK00000000, AHSL00000000, AHSM00000000, AHSN00000000, AHSO00000000, AHSP00000000, AHSQ00000000, AHSR00000000, AHSS00000000, AHST00000000, AHSU00000000, AHSV00000000, AHSW00000000, AHSX00000000, AHSY00000000, AHSZ00000000, AHTA00000000, AHTB00000000, AHTC00000000, AHTD00000000, AHTE00000000, CP003686, AP012336.

Data pre-processing for the core and pan-genome analysis were performed using a self-written perl script (Additional file 10), which is similar as described previously by Tettelin *et al*. [20]. Briefly, an iterative procedure was carried out to estimate total genes/core genes to be discovered per additional genome sequenced. The number of total genes/core genes provided by each added new genome depends on the selection of previously added genomes. All possible combinations of genomes from 1 to M (the maximal number of available genomes) were calculated. In the case more than 1000 combinations are possible, only 1000 random combinations were used. In order to take into consideration of core genes that are possibly missed during genome sequencing and assembly, for the calculation of core-genome size, an additional correction step was introduced, in which any one gene that is only absent in one of the 63 draft genomes was still regarded as core gene. During the fitting step of the core genome model, the inputted genome numbers were used as fitting weight for corresponding data point.

### Gene content-based comparative analysis of 10 mutans streptococci strains

In this work, if not otherwise specified, the uniqueness of genes from “organism A” is defined according to the ortholog groups constructed by using the OrthoMCL program [25]. If the ortholog of a gene from organism A is absent in “organism B”, we define that this gene is unique or specific to organism A in comparison to organism B. This does not imply that there is no homolog (namely paralog) of the gene from organism A in organism B. In some cases, this gene is just an additional copy of another gene whose alleles/orthologs are found in both organisms. This does further not imply that this gene is found in organism A only. For example, the ortholog of this gene may be found in organism C from the relationship table or another strain or species that is not compared in this work.

### Genome-scale metabolic networks construction

The bipartite metabolic networks were constructed based on the connection matrix of updated KEGG reactions database according to Stelzer and Zeng [97] with addition of newly identified reaction catalyzed by lactate oxidase (Lactate + O_2_ = > Pyruvate + H_2_O_2_) with provisional R numbers of R10001 (C00186 + C00007 = > C00022 + C00027) and R10002 (C00256 + C00007 = > C00022 + C00027). Compared to the reaction graph or the metabolite graph, wherein either reactions or metabolites (called "nodes") are shown in an interconnected way, the bipartite network is more comprehensible because, similar to the biochemistry textbook, both the reactions and metabolites are visualized at mean time. Seventy-six non-enzymatic automatic reactions were also considered for the network construction. The construction of sub-networks was based on the KEGG pathway classification (http://www.genome.jp/kegg/pathway.html) with slight modification of addition of reaction catalyzed by lactate oxidase into Glycolysis/Gluconeogenesis pathway (MAP00010) and Pyruvate metabolism pathway (MAP00620). The software Cytoscape [80] was used for the visualization and comparative analysis of the genome-scale metabolic networks.

### PCR verification

To verify the unique presence of the lactate oxidase (consecutive) coding genes D823_06595 and D823_06598 respectively and to exclude the possibility of contamination with e. g. human DNA during the process of genome sequencing, PCR amplification (using one primer pair covering both genes) with newly isolated DNA from *S*. *sobrinus* DSM 20742 as well as a second *S*. *sobrinus* strain (AC153) and from strains *S*. *mutans* UA159 as well as *S*. *ratti* DSM 20564 (as negative controls) was performed. The primers used were: 5′- GAGCAGGATAATTGACAGTC -3′ (forward primer) and 5′- ACTCAGTGACGAATCAGTT -3′ (reverse primer), which were designed by using Primer Premier http://www.premierbiosoft.com/primerdesign/index.html) and Vector NTI 9.0 (InforMax), respectively. Conditions for this conventional PCR were: 94°C, 2 min; followed by 32 cycles of 94°C for 30s; annealing temperature 48°C for 30s; and 72°C for 90s; final extension at 72°C for 5 min; length of amplicon 1,175 bp.

### Constructs for lactate oxidase deletion mutants and transformation of *S*. *sobrinus* DSM 20742

To clarify the functionality of the two lactate oxidases, namely D823_06598 (Llod) and D823_06595 (lod), PCR ligation mutagenesis according to the method of [98] was used to separately replace the two genes encoding the two enzymes by an erythromycin resistance cassette via double homologous recombination. Primers P1Llod (TTACCGTTATCCGCGAATTAT) and P2Llod (GGCGCGCCAACCACCCAAGGTTGAATC), P1lod (GGCTGGTTTCCTCCATGATA) and P2lod (GGCGCGCCCCAAAACCACCTTGAGGAAT) were used to amplify the 5′flanking regions of both genes, respectively, introducing an AscI restriction site. To amplify the 3′flanking regions of both genes, the primers P3Llod (GGCCGGCCGGGAGCTCAAGGTGTTCAAA) and P4Llod (CAAATTGTTCAAAGCGGGAAC), P3lod (GGCCGGCCGGCAGCAGCCGGTAGTATT) and P4lod (GGGTGCCAACTTATGTCACGA) were used, thereby introducing restriction site for FseI. The erythromycin resistance cassette was amplified from previously constructed gene deletion mutant [99] using primers ErmFor (GGCGCGCCCCGGGCCCAAAATTTGTTTGAT) and ErmRev (GGCCGGCCAGTCGGCAGCGACTCATAGAAT), containing the restriction site for AscI and FseI, respectively. After digestion with the appropriate restriction enzymes, following purification, the three amplicons were ligated together and used for transformation.

For transformation, two natural transformation methods were first used to assay and optimize the natural transformation of the *S*. *sobrinus* cells. The first step was the preparation of pre-competent cells of *S*. *sobrinus* applying the methods according to [100] and [101]. Afterwards 200 ng of constructs prepared for mutagenesis were used for the transformation. The plasmids like pDL278 (Spr, pAT18 Emr, and suicide vector pFW5 Spr in both circular and linearized form were used as a positive control. Another transformation protocol according to [102] applying pheromone CSP of *S*. *mutans* was additionally used to introduce genetic constructs and plasmids into *S*. *sobrinus* cells. In this approach two various concentrations of CSP were used: 02 and 1μM, respectively. Transformation of *S*. *mutans* was used as a parallel control. All these experiments were carried out at least three times.

Later, electroporation experiment was carried out according to the procedure described by LeBlanc *et al*. [103]. Various pHs of electroporation mix (EPM) [104] as well as various pulsing conditions were tested. The electroporation was carried out by adding to the chilled electrocompetent cells 200 ng of constructs prepared for mutagenesis or plasmids. Other protocol for electroporation according to [105] was also tested.

## Abbreviations

PCR: Polymerase Chain Reaction; PTS: Phosphotransferase system; TCA: Tricarboxylic acid cycle; ATP: adenosine-5^′^-triphosphate; PEP: Phosphoenolpyruvate; HGT: Horizontal gene transfer; LGT: Lateral gene transfer; SNP: Single-nucleotide polymorphism; LCB: Locally collinear block; multi-MUMs: Multiple maximal-unique-matches; COG: Clusters of orthologous groups of proteins; CSP: Competence stimulating peptide; XIP: Sigma X-inducing peptide; ABC transporter: ATP-binding cassette transporter; SOD: Superoxide dismutase; NAD+: Nicotinamide adenine dinucleotide; NADP+: Nicotinamide adenine dinucleotide phosphate; GSH: L-γ-glutamyl-L-cysteinylglycine; GSSG: Oxidized glutathione; GCS: Glutamylcysteine synthetase; GS: Glutathione synthetase; GCS-GS: Glutamylcysteine synthetase-glutathione synthetase; CoA: Coenzyme A.

## Competing interests

The authors declare that they have no competing interests.

## Authors’ contributions

LS carried out the bioinformatics analysis and wrote the draft. WW participated in the conception and coordination of the study and contributed significantly to results analysis and drafting the manuscript. GC provided strains with a verified identity. GC, AR, MR and IWD performed the PCR verification experiments. HS did the experiments on genomic competence of *S*. *sobrinus* and the knock-out of the two genes for lactate oxidase. GC and IWD contributed to the microbial aspects and valuable discussions. AZE conceived of and supervised the study and revised the manuscript. All authors read and approved the final manuscript.

## Supplementary Material

Additional file 1 **Presence/absence of genes related to known *S. mutans* genomic islands of 10 mutans streptococci strains.** Click here for file

Additional file 2 **Core gene list of *S. mutans.*** Click here for file

Additional file 3 Little square linear fitting details of the core and pan genome models. Click here for file

Additional file 4 Predicted ortholog groups of 10 mutans streptococci strains using OrthoMCL. Click here for file

Additional file 5 Sequences of mutacins used for the identification of putative mutacins in 10 mutans streptococci strains. Click here for file

Additional file 6 Constructed genome wide metabolic networks in Cytoscape (*.cyc) format. Click here for file

Additional file 7 **Comparative analysis of the metabolic pathways in the different metabolic networks using *S. mutans *UA159 as reference.** Absent and unique reaction numbers of metabolic networks in strains compared to *S*. *mutans* UA159. Absent and unique EC numbers of metabolic networks in strains compared to *S*. *mutans* UA159. Click here for file

Additional file 8 **PCR verifications of the unique presence of the lactate oxidase genes in *S. sobrinus *DSM 20742.** Click here for file

Additional file 9 The locations of missing genes in NCBI genome annotation results. Click here for file

Additional file 10 The perl script used for core- and pan-genome analysis in this study. Click here for file
