# Correction: Genetic variability of mutans streptococci revealed by wide whole-genome sequencing

**DOI:** 10.1186/1471-2164-14-811

**Published:** 2013-12-16

**Authors:** Lifu Song, Wei Wang, Georg Conrads, Anke Rheinberg, Helena Sztajer, Michael Reck, Irene Wagner-Döbler, An-Ping Zeng

**Affiliations:** 1 Institute of Bioprocess and Biosystems Engineering, Technical University Hamburg Harburg, Hamburg, Germany; 2 Division of Oral Microbiology and Immunology, Department of Operative and Preventive Dentistry & Periodontology, RWTH Aachen University, Aachen, Germany; 3 Department of Microbial Pathogenesis, Group Microbial Communication, Helmholtz-Centre for Infection Research, Inhoffenstrasse 7, Braunschweig, Germany

## Correction

After the publication of this work [1], we became aware of the fact that from No. 10 onwards the numbering of the references in the reference list lost congruence with citations in the body of the text. Please see below the corrected list of references. The citations in the original manuscript refer to the corrected list below.

Furthermore we like to correct two additional errors: In Figure two (Figure 1 here) the strain number KK26 has to be corrected to KK21 and on page 13 of the manuscript, chapter “Antibiotic resistance-related proteins” the unit of the minimum inhibitory concentration of *Streptococcus mutans* against bacitracin has to be changed from μg/l to μg/ml, reading now:

**Figure 1:**
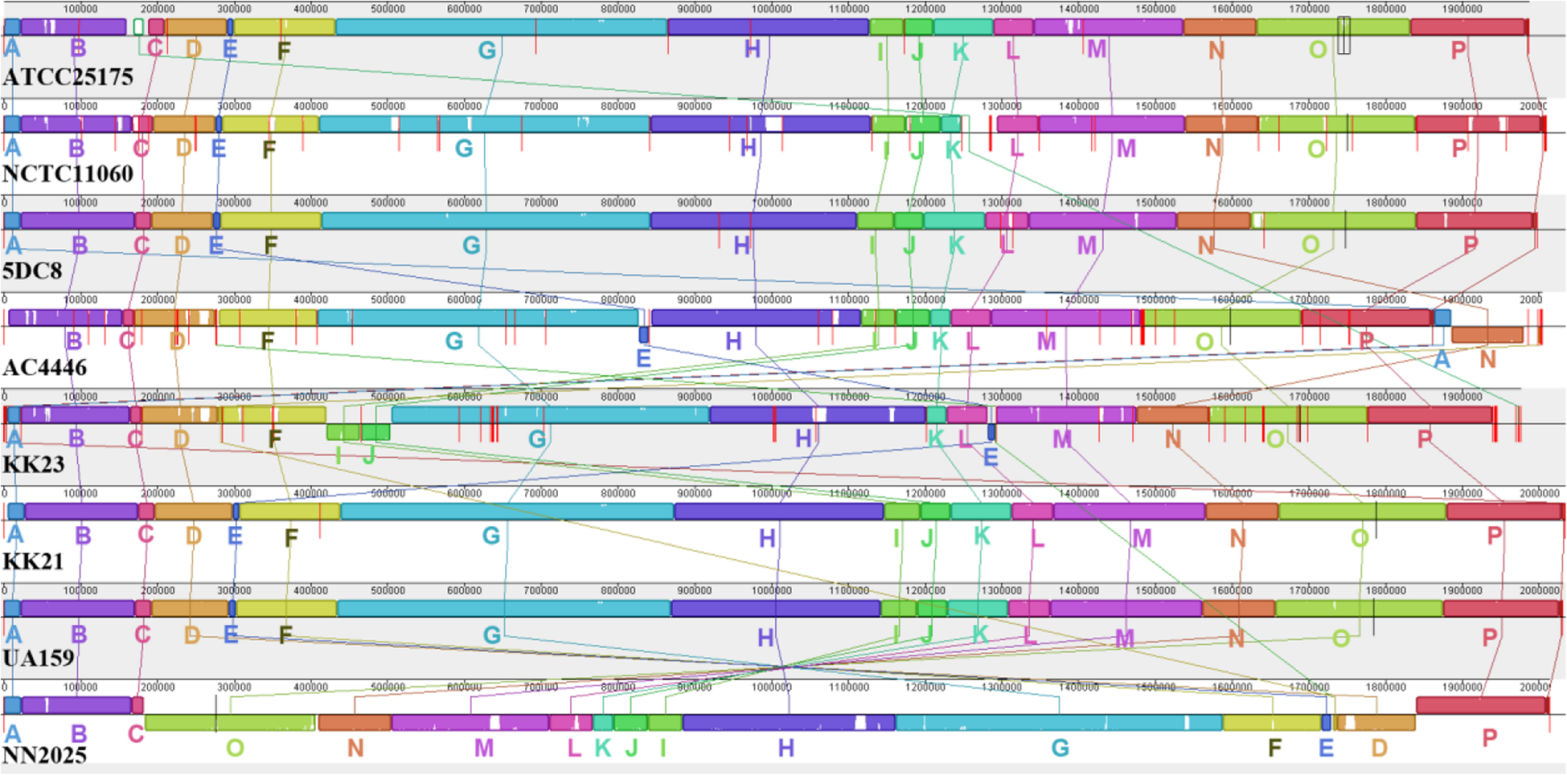
**Comparison of local collinear blocks (LCBs) of chromosomal sequences of the eight *S. mutans *strains.** In total 16 local LCBs, marked as A to P, were generated and compared by applying the MAUVE software [13,14] with default settings and using strain UA159 as reference. The red vertical bars indicate contig ends. The white areas inside each LCB show regions with low similarities.

The *S. mutans* species is known to be intrinsically resistant to bacitracin produced by *Bacillus subtilis*. We confirmed this by testing all the 10 strains with a bacitracin-E-test (data not shown). All strains including *S. ratti* DSM 20564 and *S. sobrinus* DSM 20742 had a minimum inhibitory concentration between 128 and >256 μg/ml.

We regret any inconvenience that these inaccuracies might have caused. We wish to thank Prof. Dr. Rudolf Lütticken for bringing this matter to our attention.

## References (corrected)

1. Tapp J, Thollesson M, Herrmann B: **Phylogenetic relationships and genotyping of the genus Streptococcus by sequence determination of the RNase P RNA gene, rnpB.***Int J Syst Evol Microbiol* 2003, **53:**1861**–**1871.

2. Loesche WJ: **Role of Streptococcus mutans in human dental decay.***Microbiol Rev* 1986, **50:**353**–**380.

3. Lemos JA, Burne RA: **A model of efficiency: stress tolerance by Streptococcus mutans.***Microbiology* 2008, **154:**3247**–**3255.

4. Nakano K, Nomura R, Matsumoto M, Ooshima T: **Roles of oral bacteria in cardiovascular diseases--from molecular mechanisms to clinical cases: Cell-surface structures of novel serotype k Streptococcus mutans strains and their correlation to virulence.***J Pharmacol Sci* 2010, **113:**120**–**125.

5. Nomura R, Nakano K, Taniguchi N, Lapirattanakul J, Nemoto H, Gronroos L, Alaluusua S, Ooshima T: **Molecular and clinical analyses of the gene encoding the collagen-binding adhesin of Streptococcus mutans.***J Med Microbiol* 2009, **58:**469**–**475.

6. Redfield RJ, Findlay WA, Bosse J, Kroll JS, Cameron AD, Nash JH: **Evolution of competence and DNA uptake specificity in the Pasteurellaceae.***BMC Evol Biol* 2006, **6:**82.

7. Ehrlich GD, Hu FZ, Shen K, Stoodley P, Post JC: **Bacterial plurality as a general mechanism driving persistence in chronic infections.***Clin Orthop Relat Res* 2005**:**20**–**24.

8. Ajdic D, McShan WM, McLaughlin RE, Savic G, Chang J, Carson MB, Primeaux C, Tian R, Kenton S, Jia H, et al.: **Genome sequence of Streptococcus mutans UA159, a cariogenic dental pathogen.***Proc Natl Acad Sci U S A* 2002, **99:**14434**–**14439.

9. Maruyama F, Kobata M, Kurokawa K, Nishida K, Sakurai A, Nakano K, Nomura R, Kawabata S, Ooshima T, Nakai K, et al.: **Comparative genomic analyses of Streptococcus mutans provide insights into chromosomal shuffling and species-specific content.***BMC Genomics* 2009, **10:**358.

10. Cornejo OE, Lefebure T, Pavinski Bitar PD, Lang P, Richards VP, Eilertson K, Do T, Beighton D, Zeng L, Ahn SJ, et al.: **Evolutionary and population genomics of the cavity causing bacteria Streptococcus mutans.***Mol Biol Evol* 2013, **30**:881–893.

11. Zhao Y, Wu J, Yang J, Sun S, Xiao J, Yu J: **PGAP: pan-genomes analysis pipeline.***Bioinformatics* 2012, **28:**416**–**418.

12. Darling AE, Miklos I, Ragan MA: **Dynamics of genome rearrangement in bacterial populations.***PLoS Genet* 2008, **4:**e1000128.

13. Darling AC, Mau B, Blattner FR, Perna NT: **Mauve: multiple alignment of conserved genomic sequence with rearrangements.***Genome Res* 2004, **14:**1394**–**1403.

14. Darling AE, Mau B, Perna NT: **progressive Mauve: multiple genome alignment with gene gain, loss and rearrangement.***PLoS One* 2010, **5:**e11147.

15. Waterhouse JC, Swan DC, Russell RR: **Comparative genome hybridization of Streptococcus mutans strains.***Oral Microbiol Immunol* 2007, **22:**103**–**110.

16. Wu C, Cichewicz R, Li Y, Liu J, Roe B, Ferretti J, Merritt J, Qi F: **Genomic island TnSmu2 of Streptococcus mutans harbors a nonribosomal peptide synthetase-polyketide synthase gene cluster responsible for the biosynthesis of pigments involved in oxygen and H**_**2**_**O**_**2 **_**tolerance.***Appl Environ Microbiol* 2010, **76:**5815**–**5826.

17. Waterhouse JC, Russell RR: **Dispensable genes and foreign DNA in Streptococcus mutans.***Microbiology* 2006, **152:**1777**–**1788.

18. Muzzi A, Donati C: **Population genetics and evolution of the pan-genome of Streptococcus pneumoniae.***Int J Med Microbiol* 2011, **301:**619**–**622.

19. Tettelin H, Masignani V, Cieslewicz MJ, Donati C, Medini D, Ward NL, Angiuoli SV, Crabtree J, Jones AL, Durkin AS, et al.: **Genome analysis of multiple pathogenic isolates of Streptococcus agalactiae: implications for the microbial “pan-genome”.***Proc Natl Acad Sci U S A* 2005, **102:**13950**–**13955.

20. Tettelin H, Riley D, Cattuto C, Medini D: **Comparative genomics: the bacterial pan-genome.***Curr Opin Microbiol* 2008, **11:**472**–**477.

21. Mira A, Martin-Cuadrado AB, D’Auria G, Rodriguez-Valera F: **The bacterial pan-genome: a new paradigm in microbiology.***Int Microbiol* 2010, **13:**45**–**57.

22. Donati C, Hiller NL, Tettelin H, Muzzi A, Croucher NJ, Angiuoli SV, Oggioni M, Dunning Hotopp JC, Hu FZ, Riley DR, et al.: **Structure and dynamics of the pan-genome of Streptococcus pneumoniae and closely related species.***Genome Biol* 2010, **11:**R107.

23. Lefebure T, Stanhope MJ: **Evolution of the core and pan-genome of Streptococcus: positive selection, recombination, and genome composition.***Genome Biol* 2007, **8:**R71.

24. Hogg JS, Hu FZ, Janto B, Boissy R, Hayes J, Keefe R, Post JC, Ehrlich GD: **Characterization and modeling of the Haemophilus influenzae core and supragenomes based on the complete genomic sequences of Rd and 12 clinical nontypeable strains.***Genome Biol* 2007, **8:**R103.

25. Li L, Stoeckert CJ, Jr., Roos DS: **OrthoMCL: identification of ortholog groups for eukaryotic genomes.***Genome Res* 2003, **13:**2178**–**2189.

26. Song L, Sudhakar P, Wang W, Conrads G, Brock A, Sun J, Wagner-Dobler I, Zeng AP: **A genome-wide study of two-component signal transduction systems in eight newly sequenced mutans streptococci strains.***BMC Genomics* 2012, **13:**128.

27. Tanzer JM, Livingston J, Thompson AM: **The microbiology of primary dental caries in humans.***J Dent Educ* 2001, **65:**1028**–**1037.

28. Hale JD, Heng NC, Jack RW, Tagg JR: **Identification of nlmTE, the locus encoding the ABC transport system required for export of nonlantibiotic mutacins in Streptococcus mutans.***J Bacteriol* 2005, **187:**5036**–**5039.

29. Hossain MS, Biswas I: **An extracelluar protease, SepM, generates functional competence-stimulating peptide in Streptococcus mutans UA159.***J Bacteriol* 2012, **194:**5886**–**5896.

30. Li YH, Tang N, Aspiras MB, Lau PC, Lee JH, Ellen RP, Cvitkovitch DG: **A quorum-sensing signaling system essential for genetic competence in Streptococcus mutans is involved in biofilm formation.***J Bacteriol* 2002, **184:**2699**–**2708.

31. Petersen FC, Scheie AA: **Genetic transformation in Streptococcus mutans requires a peptide secretion-like apparatus.***Oral Microbiol Immunol* 2000, **15:**329**–**334.

32. Petersen FC, Fimland G, Scheie AA: **Purification and functional studies of a potent modified quorum-sensing peptide and a two-peptide bacteriocin in Streptococcus mutans.***Mol Microbiol* 2006, **61:**1322**–**1334.

33. Allan E, Hussain HA, Crawford KR, Miah S, Ascott ZK, Khwaja MH, Hosie AH: **Genetic variation in comC, the gene encoding competence-stimulating peptide (CSP) in Streptococcus mutans.***FEMS Microbiol Lett* 2007, **268:**47**–**51.

34. Ahn SJ, Wen ZT, Burne RA: **Multilevel control of competence development and stress tolerance in Streptococcus mutans UA159.***Infect Immun* 2006, **74:**1631**–**1642.

35. Kreth J, Hung DC, Merritt J, Perry J, Zhu L, Goodman SD, Cvitkovitch DG, Shi W, Qi F: **The response regulator ComE in Streptococcus mutans functions both as a transcription activator of mutacin production and repressor of CSP biosynthesis.***Microbiology* 2007, **153:**1799**–**1807.

36. Kreth J, Merritt J, Shi W, Qi F: **Co-ordinated bacteriocin production and competence development: a possible mechanism for taking up DNA from neighbouring species.***Mol Microbiol* 2005, **57:**392**–**404.

37. Kreth J, Merritt J, Zhu L, Shi W, Qi F: **Cell density- and ComE-dependent expression of a group of mutacin and mutacin-like genes in Streptococcus mutans.***FEMS Microbiol Lett* 2006, **265:**11**–**17.

38. van der Ploeg JR: **Regulation of bacteriocin production in Streptococcus mutans by the quorum-sensing system required for development of genetic competence.***J Bacteriol* 2005, **187:**3980**–**3989.

39. Mashburn-Warren L, Morrison DA, Federle MJ: **A novel double-tryptophan peptide pheromone controls competence in Streptococcus spp. via an Rgg regulator.***Mol Microbiol* 2010, **78:**589**–**606.

40. Okinaga T, Niu G, Xie Z, Qi F, Merritt J: **The hdrRM operon of Streptococcus mutans encodes a novel regulatory system for coordinated competence development and bacteriocin production.***J Bacteriol* 2010, **192:**1844**–**1852.

41. Okinaga T, Xie Z, Niu G, Qi F, Merritt J: **Examination of the hdrRM regulon yields insight into the competence system of Streptococcus mutans.***Mol Oral Microbiol* 2010, **25:**165**–**177.

42. Xie Z, Okinaga T, Niu G, Qi F, Merritt J: **Identification of a novel bacteriocin regulatory system in Streptococcus mutans.***Mol Microbiol* 2010, **78:**1431**–**1447.

43. Mair RW, Senadheera DB, Cvitkovitch DG: **CinA is regulated via ComX to modulate genetic transformation and cell viability in Streptococcus mutans.***FEMS Microbiol Lett* 2012, **331:**44**–**52.

44. Alaluusua S, Takei T, Ooshima T, Hamada S: **Mutacin activity of strains isolated from children with varying levels of mutants streptococci and caries.***Arch Oral Biol* 1991, **36:**251**–**255.

45. Baba T, Schneewind O: **Instruments of microbial warfare: bacteriocin synthesis, toxicity and immunity.***Trends Microbiol* 1998, **6:**66**–**71.

46. Hossain MS, Biswas I: **Mutacins from Streptococcus mutans UA159 are active against multiple streptococcal species.***Appl Environ Microbiol* 2011, **77:**2428**–**2434.

47. Yonezawa H, Kuramitsu HK: **Genetic analysis of a unique bacteriocin, Smb, produced by Streptococcus mutans GS5.***Antimicrob Agents Chemother* 2005, **49:**541**–**548.

48. Hyink O, Balakrishnan M, Tagg JR: **Streptococcus rattus strain BHT produces both a class I two-component lantibiotic and a class II bacteriocin.***FEMS Microbiol Lett* 2005, **252:**235**–**241.

49. Nguyen T, Zhang Z, Huang IH, Wu C, Merritt J, Shi W, Qi F: **Genes involved in the repression of mutacin I production in Streptococcus mutans.***Microbiology* 2009, **155:**551**–**556.

50. Qi F, Chen P, Caufield PW: **Purification and biochemical characterization of mutacin I from the group I strain of Streptococcus mutans, CH43, and genetic analysis of mutacin I biosynthesis genes.***Appl Environ Microbiol* 2000, **66:**3221**–**3229.

51. Chen P, Qi F, Novak J, Caufield PW: **The specific genes for lantibiotic mutacin II biosynthesis in Streptococcus mutans T8 are clustered and can be transferred en bloc.***Appl Environ Microbiol* 1999, **65:**1356**–**1360.

52. Qi F, Chen P, Caufield PW: **Purification of mutacin III from group III Streptococcus mutans UA787 and genetic analyses of mutacin III biosynthesis genes.***Appl Environ Microbiol* 1999, **65:**3880**–**3887.

53. Robson CL, Wescombe PA, Klesse NA, Tagg JR: **Isolation and partial characterization of the Streptococcus mutans type AII lantibiotic mutacin K8.***Microbiology* 2007, **153:**1631**–**1641.

54. Qi F, Chen P, Caufield PW: **The group I strain of Streptococcus mutans, UA140, produces both the lantibiotic mutacin I and a nonlantibiotic bacteriocin, mutacin IV.***Appl Environ Microbiol* 2001, **67:**15**–**21.

55. Hale JD, Ting YT, Jack RW, Tagg JR, Heng NC: **Bacteriocin (mutacin) production by Streptococcus mutans genome sequence reference strain UA159: elucidation of the antimicrobial repertoire by genetic dissection.***Appl Environ Microbiol* 2005, **71:**7613**–**7617.

56. Dufour D, Cordova M, Cvitkovitch DG, Levesque CM: **Regulation of the competence pathway as a novel role associated with a streptococcal bacteriocin.***J Bacteriol* 2011, **193:**6552**–**6559.

57. Nes IF, Diep DB, Holo H: **Bacteriocin diversity in Streptococcus and Enterococcus.***J Bacteriol* 2007, **189:**1189**–**1198.

58. Bekal-Si Ali S, Hurtubise Y, Lavoie MC, LaPointe G: **Diversity of Streptococcus mutans bacteriocins as confirmed by DNA analysis using specific molecular probes.***Gene* 2002, **283:**125**–**131.

59. Johnson DW, Tagg JR, Wannamaker LW: **Production of a bacteriocine-like substance by group-A streptococci of M-type 4 and T-pattern 4.***J Med Microbiol* 1979, **12:**413**–**427.

60. Perry JA, Jones MB, Peterson SN, Cvitkovitch DG, Levesque CM: **Peptide alarmone signalling triggers an auto-active bacteriocin necessary for genetic competence.***Mol Microbiol* 2009, **72:**905**–**917.

61. Chatterjee AK, Starr MP: **Transfer among Erwinia spp. and other enterobacteria of antibiotic resistance carried on R factors.***J Bacteriol* 1972, **112:**576**–**584.

62. Yano H, Kuga A, Okamoto R, Kitasato H, Kobayashi T, Inoue M: **Plasmid-encoded metallo-beta-lactamase (IMP-6) conferring resistance to carbapenems, especially meropenem.***Antimicrob Agents Chemother* 2001, **45:**1343**–**1348.

63. Ouyang J, Tian XL, Versey J, Wishart A, Li YH: **The BceABRS four-component system regulates the bacitracin-induced cell envelope stress response in Streptococcus mutans.***Antimicrob Agents Chemother* 2010, **54:**3895**–**3906.

64. Tsuda H, Yamashita Y, Shibata Y, Nakano Y, Koga T: **Genes involved in bacitracin resistance in Streptococcus mutans.***Antimicrob Agents Chemother* 2002, **46:**3756**–**3764.

65. El Ghachi M, Bouhss A, Blanot D, Mengin-Lecreulx D: **The bacA gene of Escherichia coli encodes an undecaprenyl pyrophosphate phosphatase activity.***J Biol Chem* 2004, **279:**30106**–**30113.

66. Bernard R, El Ghachi M, Mengin-Lecreulx D, Chippaux M, Denizot F: **BcrC from Bacillus subtilis acts as an undecaprenyl pyrophosphate phosphatase in bacitracin resistance.***J Biol Chem* 2005, **280:**28852**–**28857.

67. McCord JM, Fridovich I: **Superoxide dismutase: the first twenty years (1968–1988).***Free Radic Biol Med* 1988, **5:**363**–**369.

68. Yamamoto Y, Higuchi M, Poole LB, Kamio Y: **Role of the dpr product in oxygen tolerance in Streptococcus mutans.***J Bacteriol* 2000, **182:**3740**–**3747.

69. Higuchi M, Yamamoto Y, Kamio Y: **Molecular biology of oxygen tolerance in lactic acid bacteria: Functions of NADH oxidases and Dpr in oxidative stress.***J Biosci Bioeng* 2000, **90:**484**–**493.

70. Yamamoto Y, Poole LB, Hantgan RR, Kamio Y: **An iron-binding protein, Dpr, from Streptococcus mutans prevents iron-dependent hydroxyl radical formation in vitro.***J Bacteriol* 2002, **184:**2931**–**2939.

71. Mustacich D, Powis G: **Thioredoxin reductase.***Biochem J* 2000, **346 Pt 1:**1**–**8.

72. Arner ES, Holmgren A: **Physiological functions of thioredoxin and thioredoxin reductase.***Eur J Biochem* 2000, **267:**6102**–**6109.

73. Seo HJ, Lee YN: **Characterization of Deinococcus radiophilus thioredoxin reductase active with both NADH and NADPH.***J Microbiol* 2010, **48:**637**–**643.

74. Holmgren A: **Thioredoxin and glutaredoxin systems.***J Biol Chem* 1989, **264:**13963**–**13966.

75. Fernandes AP, Holmgren A: **Glutaredoxins: glutathione-dependent redox enzymes with functions far beyond a simple thioredoxin backup system.***Antioxid Redox Signal* 2004, **6:**63**–**74.

76. Fahey RC, Brown WC, Adams WB, Worsham MB: **Occurrence of glutathione in bacteria.***J Bacteriol* 1978, **133:**1126**–**1129.

77. Janowiak BE, Griffith OW: **Glutathione synthesis in Streptococcus agalactiae. One protein accounts for gamma-glutamylcysteine synthetase and glutathione synthetase activities.***J Biol Chem* 2005, **280:**11829**–**11839.

78. Zhang J, Biswas I: **3′-Phosphoadenosine-5′-phosphate phosphatase activity is required for superoxide stress tolerance in Streptococcus mutans.***J Bacteriol* 2009, **191:**4330**–**4340.

79. Ma H, Zeng AP: **Reconstruction of metabolic networks from genome data and analysis of their global structure for various organisms.***Bioinformatics* 2003, **19:**270**–**277.

80. Kohl M, Wiese S, Warscheid B: **Cytoscape: software for visualization and analysis of biological networks.***Methods Mol Biol* 2011, **696:**291**–**303.

81. Taniai H, Iida K, Seki M, Saito M, Shiota S, Nakayama H, Yoshida S: **Concerted action of lactate oxidase and pyruvate oxidase in aerobic growth of Streptococcus pneumoniae: role of lactate as an energy source.***J Bacteriol* 2008, **190:**3572**–**3579.

82. Kreth J, Merritt J, Shi W, Qi F: **Competition and coexistence between Streptococcus mutans and Streptococcus sanguinis in the dental biofilm.***J Bacteriol* 2005, **187:**7193**–**7203.

83. Okahashi N, Nakata M, Sumitomo T, Terao Y, Kawabata S: **Hydrogen peroxide produced by oral streptococci induces macrophage cell death.***PLoS One* 2013, **8:**e62563.

84. Subramanian S, Sivaraman C: **Bacterial citrate lyase.***Journal of Biosciences* 1984, **6:**379–401.

85. Evans HJ, Wood HG: **The mechanism of the pyruvate, phosphate dikinase reaction.***Proc Natl Acad Sci U S A* 1968, **61:**1448**–**1453.

86. Benziman M, Eisen N, Palgi A: **Properties and physiological role of the pep-synthase of A. xylinum.***FEBS Lett* 1969, **3:**156**–**159.

87. Sauer U, Eikmanns BJ: **The PEP-pyruvate-oxaloacetate node as the switch point for carbon flux distribution in bacteria.***FEMS Microbiol Rev* 2005, **29:**765**–**794.

88. Cvitkovitch DG, Gutierrez JA, Bleiweis AS: **Role of the citrate pathway in glutamate biosynthesis by Streptococcus mutans.***J Bacteriol* 1997, **179:**650**–**655.

89. Chain PS, Grafham DV, Fulton RS, Fitzgerald MG, Hostetler J, Muzny D, Ali J, Birren B, Bruce DC, Buhay C, et al.: **Genomics. Genome project standards in a new era of sequencing.***Science* 2009, **326:**236**–**237.

90. Li R, Yu C, Li Y, Lam TW, Yiu SM, Kristiansen K, Wang J: **SOAP2: an improved ultrafast tool for short read alignment.***Bioinformatics* 2009, **25:**1966**–**1967.

91. Li H, Durbin R: **Fast and accurate short read alignment with Burrows-Wheeler transform.***Bioinformatics* 2009, **25:**1754**–**1760.

92. de la Bastide M, McCombie WR: **Assembling genomic DNA sequences with PHRAP.***Curr Protoc Bioinformatics* 2007, **Chapter 11:**Unit11 14.

93. Rissman AI, Mau B, Biehl BS, Darling AE, Glasner JD, Perna NT: **Reordering contigs of draft genomes using the Mauve aligner.***Bioinformatics* 2009, **25:**2071**–**2073.

94. Delcher AL, Bratke KA, Powers EC, Salzberg SL: **Identifying bacterial genes and endosymbiont DNA with Glimmer.***Bioinformatics* 2007, **23:**673**–**679.

95. Conesa A, Gotz S, Garcia-Gomez JM, Terol J, Talon M, Robles M: **Blast2GO: a universal tool for annotation, visualization and analysis in functional genomics research.***Bioinformatics* 2005, **21:**3674**–**3676.

96. Karp PD, Paley S, Romero P: **The Pathway Tools software.***Bioinformatics* 2002, **18 Suppl 1:**S225-232.

97. Stelzer M, Sun J, Kamphans T, Fekete SP, Zeng AP: **An extended bioreaction database that significantly improves reconstruction and analysis of genome-scale metabolic networks.***Integr Biol (Camb)* 2011, **3:**1071**–**1086.

98. Lau PC, Sung CK, Lee JH, Morrison DA, Cvitkovitch DG: **PCR ligation mutagenesis in transformable streptococci: application and efficiency.***J Microbiol Methods* 2002, **49:**193**–**205.

99. Reck M, Rutz K, Kunze B, Tomasch J, Surapaneni SK, Schulz S, Wagner-Dobler I: **The biofilm inhibitor carolacton disturbs membrane integrity and cell division of Streptococcus mutans through the serine/threonine protein kinase PknB.***J Bacteriol* 2011, **193:**5692**–**5706.

100. Lefrancois J, Samrakandi MM, Sicard AM: **Electrotransformation and natural transformation of Streptococcus pneumoniae: requirement of DNA processing for recombination.***Microbiology* 1998, **144 (Pt 11):**3061**–**3068.

101. Ween O, Teigen S, Gaustad P, Kilian M, Havarstein LS: **Competence without a competence pheromone in a natural isolate of Streptococcus infantis.***J Bacteriol* 2002, **184:**3426**–**3432.

102. Li YH, Lau PC, Lee JH, Ellen RP, Cvitkovitch DG: **Natural genetic transformation of Streptococcus mutans growing in biofilms.***J Bacteriol* 2001, **183:**897**–**908.

103. LeBlanc D, Chen Y-Y, Buckley N, Lee L: **Genetic transfer methods for Streptococcus sobrinus and other oral streptococci.***Methods in Cell Science* 1998, **20:**85**–**93.

104. Caparon MG, Scott JR: **Genetic manipulation of pathogenic streptococci.***Methods Enzymol* 1991, **204:**556**–**586.

105. McLaughlin RE, Ferretti JJ: **Electrotransformation of Streptococci.***Methods Mol Biol* 1995, **47:**185**–**193.
