# Supplementary material for: Genetic variability of mutans streptococci revealed by wide whole-genome sequencing
Source: BMC Genomics. 2013 Jun 28;14:430. doi: 10.1186/1471-2164-14-430 (PMC3751929; doi:10.1186/1471-2164-14-430)
Supplement: Additional file 3 — Little square linear fitting details of the core and pan genome models. [file 1471-2164-14-430-S3.docx]

## Core-genome modeling of *S. mutans*:

$F_{c}\left( n \right)=\kappa_{c}\exp\left\lfloor\frac{-n}{\tau_{c}} \right\rfloor+\Omega$

### Fitting results using all medians:

$\Omega$ = 1369.41225 (Standard Error = 1.986)

$\kappa_{c}$ = 325.74718 (Standard Error = 10.00912)

$\tau_{c}$ = 15.90248 (Standard Error = 0.66807)

R^2^ = 0.97403

Chi-Sqr tolerance value of 1E-9 was reached.

## Pan-genome modeling of *S. mutans*:

### Fitting results of model $\boldsymbol{y}\boldsymbol{=a+b}\boldsymbol{x}^{\boldsymbol{c}}$:

$a$ = 1093.74502 (Standard Error = 7.59549)

*b* = 840.62477 (Standard Error = 5.94216)

*c =* 0.31778 (Standard Error = 0.00116)

R^2^ = 0.99998

Chi-Sqr tolerance value of 1E-9 was reached.

### Fitting results of model $\boldsymbol{y}\boldsymbol{=a-bln}\left( \boldsymbol{x+c} \right)$:

$a$ = 186.56216 (Standard Error = 56.14043)

*b* = -950.12142 (Standard Error = 13.07882)

*c =* 5.94019 (Standard Error = 0.2999)

R^2^ = 0.99874

Chi-Sqr tolerance value of 1E-9 was reached.

### Fitting results of model $\boldsymbol{y}\boldsymbol{=a\times}\boldsymbol{e}^{\boldsymbol{-}\boldsymbol{x}\boldsymbol{/b}}\boldsymbol{+}\boldsymbol{c}$:

$a$ = -2383.52135 (Standard Error = 31.14844)

*b* = 30.19285 (Standard Error = 1.25161)

*c =* 4482.55361 (Standard Error = 38.11749)

R^2^ = 0.99257

Chi-Sqr tolerance value of 1E-9 was reached.
