# Supplementary material for: Genetic variability of mutans streptococci revealed by wide whole-genome sequencing
Source: BMC Genomics. 2013 Jun 28;14:430. doi: 10.1186/1471-2164-14-430 (PMC3751929; doi:10.1186/1471-2164-14-430)
Supplement: Additional file 5 — Sequences of mutacins used for the identification of putative mutacins in 10 mutans streptococci strains. [file 1471-2164-14-430-S5.docx]

**>SmbA**

MKSNLLKINNVTEMEKNMVTLIKDEDMLAGGSTPACAIGVVGITVAVTGISTACTSRCINK

**>SmbB**

MKEIQKAGLQEELSILMDDANNLEQLTAGIGTTVVNSTFSIVLGNKGYICTVTVECMRNCSK

**>Mutacin-I   (Isolated from strain UA140  AND CH43)**

MSNTQLLEVLGTETFDVQEDLFAFDTTDTTIVASNDDPDTRFSSLSLCSLGCTGVKNPSFNSYCC

**>Mutacin-II AAC38144.1**

MNKLNSNAVVSLNEVSDSELDTILGGNRWWQGVVPTVSYECRMN

**>Mut-III  (Isolated from strain UA787 1140)**

MSNTQLLEVLGTETFDVQEDLFAFDTTDTTIVASNDDPDTRFKSWSLCTPGCARTGSFNSYCC

**>Mutacin-IV SMU.150 nlmA  non-lantibiotic mutacin IV A**

MDTQAFEQFDVMDSQTLSTVEGGKVSGGEAVAAIGICATASAAIGGLAGA

TLVTPYCVGTWGLIRSH

**>Mutacin-IV SMU.151 nlmB non-lantibiotic mutacin IV B**

MEWRINTMELNVNNYKSLTNDELSEVFGGDKQAADTFLSAVGGAASGFTY

CASNGVWHPYILAGCAGVGAVGSVVFPH

**>Mutacin-V SMU.1914c CipB**

MNTQAFEQFNVMDNEALSAVEGGGRGWNCAAGIALGAGQGYMATAGGTAF

LGPYAIGTGAFGAIAGGIGGALNSCG

**>SMU.423 possible bacteriocin Kreth et al., 2005, state that this mutacin-like gene is regulated by the competence system.**

MNTQAFEQFNVMDNEALSTVEGGGMIRCALGTAGSAGLGFVGGMGAGTVT

LPVVGTVSGAALGGWSGAAVGAATF

**>Mutacin-AII (S. pyo FF22, homolog found in *S.mutans*)**

MEKNNEVINSIQEVSLEELDQIIGAGKNGVFKTISHECHLNTWA
