# Supplementary material for: Genetic variability of mutans streptococci revealed by wide whole-genome sequencing
Source: BMC Genomics. 2013 Jun 28;14:430. doi: 10.1186/1471-2164-14-430 (PMC3751929; doi:10.1186/1471-2164-14-430)
Supplement: Additional file 7 — Comparative analysis of the metabolic pathways in the different metabolic networks using S. mutans UA159 as reference. Absent and unique reaction numbers of metabolic networks in strains compared to S. mutans UA159. Absent and unique EC numbers of metabolic networks in strains compared to S. mutans UA159. [file 1471-2164-14-430-S7.docx]

Comparative analysis of the metabolic pathways in the different metabolic networks using *S. mutans* UA159 as reference.

The pathways were classified based on the KEGG pathway classification (<http://www.genome.jp/kegg/pathway.html>).

The numbers stand for the numbers of absent or unique reactions in the corresponding pathways. The R numbers (unique reaction identifiers of KEGG) and EC numbers of the absent and unique reactions are given in the **Additional file 11** and **Additional file 12**, respectively.

|  | ***S. mutans***  **NN2025** | | ***S. mutans***  **5DC8& KK21** | | ***S. mutans***  **AC4446** | | ***S. mutans***  **ATCC 25175** | | ***S. mutans***  **NCTC 11060** | | ***S. ratti***  **DSM 20564** | | ***S. sobrinus***  **DSM 20742** | |
| --- | --- | --- | --- | --- | --- | --- | --- | --- | --- | --- | --- | --- | --- | --- |
|  | Absent | Unique | Absent | Unique | Absent | Unique | Absent | Unique | Absent | Unique | Absent | Unique | Absent | Unique |
| Metabolic pathways | 2 | 5 | 1 | 21 | 2 | 0 | 2 | 16 | 0 | 16 | 4 | 16 | 13 | 5 |
| **Citrate cycle (TCA cycle)** | **0** | **0** | **0** | **0** | **0** | **0** | **0** | **0** | **0** | **0** | **0** | **0** | **1** | **0** |
| **Glycolysis / Gluconeogenesis** | **0** | **0** | **0** | **0** | **0** | **0** | **0** | **0** | **0** | **0** | **0** | **1** | **0** | **2** |
| Drug metabolism - other enzymes | 0 | 0 | 0 | 0 | 0 | 0 | 0 | 0 | 0 | 0 | 3 | 0 | 3 | 0 |
| **Fatty acid elongation** | **0** | **0** | **0** | **7** | **0** | **0** | **0** | **7** | **0** | **7** | **0** | **0** | **0** | **0** |
| Toluene degradation | 0 | 0 | 0 | 1 | 0 | 0 | 0 | 1 | 0 | 1 | 0 | 0 | 0 | 0 |
| Glycerolipid metabolism | 0 | 0 | 0 | 0 | 0 | 0 | 0 | 0 | 0 | 0 | 0 | 1 | 1 | 0 |
| Pentose phosphate pathway | 0 | 0 | 0 | 0 | 0 | 0 | 0 | 0 | 0 | 0 | 1 | 2 | 1 | 0 |
| Starch and sucrose metabolism | 0 | 0 | 0 | 0 | 0 | 0 | 0 | 0 | 0 | 0 | 0 | 0 | 1 | 0 |
| **Alanine, aspartate and glutamate metabolism** | **1** | **0** | **0** | **0** | **0** | **0** | **0** | **0** | **0** | **0** | **0** | **1** | **2** | **0** |
| alpha-Linolenic acid metabolism | 0 | 0 | 0 | 3 | 0 | 0 | 0 | 3 | 0 | 3 | 0 | 0 | 0 | 0 |
| Pentose and glucuronate inter conversions | 0 | 0 | 0 | 0 | 0 | 0 | 0 | 0 | 0 | 0 | 1 | 2 | 2 | 0 |
| **Caprolactam degradation** | **0** | **0** | **0** | **1** | **0** | **0** | **0** | **1** | **0** | **1** | **0** | **0** | **0** | **0** |
| Butanoate metabolism | 0 | 0 | 0 | 1 | 0 | 0 | 0 | 1 | 0 | 1 | 0 | 0 | 0 | 0 |
| Fructose and mannose metabolism | 0 | 0 | 0 | 0 | 0 | 0 | 0 | 0 | 0 | 0 | 1 | 2 | 1 | 0 |
| Sphingolipid metabolism | 0 | 0 | 0 | 0 | 0 | 0 | 0 | 0 | 0 | 0 | 0 | 0 | 1 | 0 |
| Primary bile acid biosynthesis | 0 | 0 | 0 | 2 | 0 | 0 | 0 | 2 | 0 | 2 | 0 | 0 | 0 | 0 |
| Glycine, serine and threonine metabolism | 0 | 0 | 0 | 0 | 0 | 0 | 0 | 0 | 0 | 0 | 0 | 0 | 0 | 2 |
| **Pyruvate metabolism** | **1** | **0** | **0** | **0** | **1** | **0** | **0** | **0** | **0** | **0** | **0** | **1** | **0** | **2** |
| **Biosynthesis of secondary metabolites** | **0** | **2** | **0** | **4** | **0** | **0** | **0** | **2** | **0** | **2** | **5** | **2** | **7** | **2** |
| Purine metabolism | 1 | 1 | 1 | 0 | 1 | 0 | 0 | 0 | 0 | 1 | 0 | 1 | 0 | 1 |
| **Galactose metabolism** | **0** | **0** | **0** | **0** | **0** | **0** | **0** | **0** | **0** | **0** | **0** | **0** | **9** | **0** |
| **Microbial metabolism in diverse environments** | **2** | **0** | **1** | **3** | **2** | **0** | **0** | **3** | **0** | **3** | **3** | **4** | **6** | **1** |
| Porphyrin and chlorophyll metabolism | 0 | 0 | 0 | 0 | 0 | 0 | 0 | 0 | 0 | 0 | 1 | 0 | 1 | 0 |
| Nitrogen metabolism | 3 | 0 | 3 | 0 | 3 | 0 | 0 | 0 | 0 | 0 | 0 | 1 | 2 | 0 |
| Lysine degradation | 0 | 2 | 0 | 3 | 0 | 0 | 0 | 1 | 0 | 1 | 0 | 0 | 0 | 0 |
| C5-Branched dibasic acid metabolism | 0 | 0 | 0 | 0 | 0 | 0 | 0 | 0 | 0 | 0 | 0 | 0 | 1 | 0 |
| Taurine and hypotaurine metabolism | 0 | 0 | 0 | 0 | 0 | 0 | 0 | 0 | 0 | 0 | 0 | 1 | 0 | 0 |
| Lysine biosynthesis | 0 | 3 | 0 | 3 | 0 | 0 | 0 | 0 | 0 | 0 | 0 | 0 | 0 | 0 |
| Arginine and proline metabolism | 2 | 0 | 2 | 0 | 2 | 0 | 0 | 0 | 0 | 0 | 0 | 1 | 1 | 0 |
| Vitamin B6 metabolism | 0 | 0 | 0 | 0 | 0 | 0 | 0 | 0 | 0 | 0 | 0 | 1 | 0 | 1 |
| Carbon fixation pathways in prokaryotes | 0 | 0 | 0 | 1 | 0 | 0 | 0 | 1 | 0 | 1 | 0 | 0 | 1 | 0 |
| Drug metabolism - cytochrome P450 | 0 | 0 | 0 | 0 | 0 | 0 | 0 | 0 | 0 | 0 | 2 | 0 | 2 | 0 |
| Amino sugar and nucleotide sugar metabolism | 0 | 0 | 0 | 0 | 0 | 0 | 0 | 0 | 0 | 0 | 0 | 0 | 4 | 0 |
| Tropane, piperidine and pyridine alkaloid biosynthesis | 0 | 0 | 0 | 0 | 0 | 0 | 0 | 0 | 0 | 0 | 1 | 0 | 1 | 0 |
| Glycosphingolipid biosynthesis - globo series | 0 | 0 | 0 | 0 | 0 | 0 | 0 | 0 | 0 | 0 | 0 | 0 | 1 | 0 |
| Valine, leucine and isoleucine degradation | 0 | 0 | 0 | 2 | 0 | 0 | 0 | 2 | 0 | 2 | 0 | 0 | 0 | 0 |
| Carbon fixation in photosynthetic organisms | 1 | 0 | 0 | 0 | 1 | 0 | 0 | 0 | 0 | 0 | 0 | 4 | 0 | 0 |
| Glycerophospholipid metabolism | 0 | 0 | 0 | 0 | 0 | 0 | 0 | 0 | 0 | 0 | 0 | 2 | 0 | 0 |
| Pyrimidine metabolism | 0 | 0 | 0 | 0 | 0 | 0 | 2 | 0 | 0 | 0 | 0 | 0 | 0 | 0 |
| Geraniol degradation | 0 | 0 | 0 | 1 | 0 | 0 | 0 | 1 | 0 | 1 | 0 | 0 | 0 | 0 |
| Ascorbate and aldarate metabolism | 0 | 0 | 0 | 0 | 0 | 0 | 0 | 0 | 0 | 0 | 0 | 2 | 1 | 0 |
| Glutathione metabolism | 0 | 0 | 0 | 0 | 0 | 0 | 0 | 0 | 0 | 0 | 0 | 0 | 2 | 0 |
| Riboflavin metabolism | 0 | 0 | 0 | 0 | 0 | 0 | 0 | 0 | 0 | 0 | 0 | 3 | 0 | 0 |
| Tryptophan metabolism | 0 | 0 | 0 | 1 | 0 | 0 | 0 | 1 | 0 | 1 | 0 | 0 | 0 | 0 |
| Terpenoid backbone biosynthesis | 0 | 0 | 0 | 0 | 0 | 0 | 0 | 0 | 0 | 0 | 2 | 0 | 1 | 1 |
| Methane metabolism | 0 | 0 | 0 | 0 | 0 | 0 | 0 | 0 | 0 | 0 | 2 | 2 | 2 | 0 |

Additional file 11. Absent and unique reaction numbers of metabolic networks in strains compared to *S. mutans* UA159.

| ***S. mutans***  **NN2025** | | ***S. mutans***  **5DC8** | | ***S. mutans***  **KK21** | | ***S. mutans***  **KK23** | | ***S. mutans***  **AC4446** | | ***S. mutans***  **ATCC25175** | | ***S. mutans***  **NCTC11060** | | ***S. ratti***  **DSM20564** | | ***S. sobrinus***  **DMS20742** | |
| --- | --- | --- | --- | --- | --- | --- | --- | --- | --- | --- | --- | --- | --- | --- | --- | --- | --- |
| Unique | Absent | Unique | Absent | Unique | Absent | Unique | Absent | Unique | Absent | Unique | Absent | Unique | Absent | Unique | Absent | Unique | Absent |
| R03102  R03098  R01856  R04390  R04863  R03103 | R00150  R01399  R00214  R01416  R00491  R06138  R01395 |  |  |  |  | R05575  R03102  R03098  R05066  R04739  R04741  R04390  R04743  R07890  R01975  R06941  R04810  R04863  R04737  R07898  R08094  R04745  R04812  R01778  R04203  R07894  R03103  R04748 | R00150  R01399  R01416  R06138  R01395 | R03102  R03098  R04390  R04863  R03103 | R00150  R01399  R00214  R01416  R06138  R01395 | R05575  R05066  R04739  R04741  R04743  R07890  R01975  R06941  R04810  R04737  R07898  R08094  R04745  R04812  R01778  R04203  R07894  R04748 | R00573  R00571 | R05575  R05066  R04739  R01856  R04741  R04743  R07890  R01975  R06941  R04810  R04737  R07898  R08094  R04745  R04812  R02364  R01778  R04203  R07894  R04748 |  | R07281  R00552  R01621  R07607  R06132  R01556  R00091  R00847  R00425  R00749  R00307  R00365  R00762  R01967  R00206  R08717  R02752  R06251  R00066  R01430  R07175  R00505  R00856  R01845  R00396  R05681  R08056  R07145  R04780  R00878  R01432  R08718  R05837 | R08575  R07140  R00868  R08258  R05823  R00527  R08255  R01827  R06728  R05604  R06983  R08295  R02081  R08300  R00630  R04496  R02082  R08220  R08878  R00310 | R07607  R00751  R01556  R01967  R06171  R06251  R00505  R05636  R05681  R02364  R00319  R07145  R05837  R10001  R10002 | R00362  R08575  R00354  R07140  R01103  R03634  R06091  R08360  R00868  R00471  R08258  R01092  R00995  R06079  R03618  R05549  R01323  R00527  R00414  R08255  R05850  R00420  R09675  R06096  R04019  R01827  R07180  R06728  R05604  R02707  R06152  R06093  R00114  R06142  R06983  R04470  R08295  R03448  R01104  R06094  R01329  R08300  R00630  R06070  R01194  R08220  R02926  R08362  R05140  R05961  R00093  R00310  R05634  R01101  R04449  R00310  R05634  R01101  R04449 |

Additional file 12. Absent and unique EC numbers of metabolic networks in strains compared to *S. mutans* UA159.

| ***S. mutans***  **NN2025** | | ***S. mutans***  **5DC8** | | ***S. mutans***  **KK21** | | ***S. mutans***  **KK23** | | ***S. mutans***  **AC4446** | | ***S. mutans***  **ATCC25175** | | ***S. mutans***  **NCTC11060** | | ***S. ratti***  **DSM20564** | | ***S. sobrinus***  **DMS20742** | |
| --- | --- | --- | --- | --- | --- | --- | --- | --- | --- | --- | --- | --- | --- | --- | --- | --- | --- |
| Unique | Absent | Unique | Absent | Unique | Absent | Unique | Absent | Unique | Absent | Unique | Absent | Unique | Absent | Unique | Absent | Unique | Absent |
| 1.2.1.31  3.1.5.1  3.6.1.3 | 2.7.2.2  5.1.1.13  3.1.21.3  3.5.3.12  1.1.1.38  2.1.3.6  4.1.1.0 |  | 3.1.21.3 |  | 2.7.7.49 | 1.1.1.35  1.2.1.31  3.6.1.3 | 2.7.2.2  3.1.21.3  3.5.3.12  2.7.7.49  2.1.3.6 | 1.2.1.31  3.6.1.3 | 2.7.2.2  3.1.21.3  3.5.3.12  1.1.1.38  3.1.21.4  2.1.3.6  4.1.1.0 | 1.1.1.35  3.6.1.3 | 6.3.4.2  3.1.21.3  2.7.7.49 | 1.1.1.35  1.6.5.5  3.1.5.1  3.6.1.3 | 3.1.21.3  2.7.7.49 | 1.1.1.0  1.1.1.14  1.1.1.179  1.1.1.262  1.4.1.1  1.8.1.14  1.8.4.12  2.3.1.79  2.5.1.9  2.7.1.113  2.7.1.30  2.7.7.39  2.7.9.1  3.1.3.11  3.2.1.24  3.5.3.6  3.5.4.25  3.6.3.3  3.6.3.5  4.1.2.9  4.1.99.12  4.2.1.40  4.3.1.7  5.3.1.5  5.4.99.9 | 3.6.3.28  3.1.2.12  1.1.1.274  4.99.1.1  1.1.1.88  3.1.21.3  1.1.1.11  2.7.7.49  3.1.1.0  3.1.21.4  1.1.1.34  3.1.2.0  1.1.1.284  2.2.1.2  2.1.1.100  3.1.1.1 | 1.13.12.4  1.1.1.14  1.1.1.262  1.6.5.5  1.8.4.12  2.2.1.7  2.3.1.79  2.4.2.22  2.7.1.113  3.6.1.3  3.6.3.3  3.6.3.5  4.1.2.5  5.4.99.9 | 3.6.3.28  5.1.3.4  3.1.2.12  4.1.1.3  4.99.1.1  2.4.1.10  5.3.1.0  1.1.1.11  3.2.2.21  2.7.8.25  2.7.7.49  3.1.1.0  1.11.1.15  2.8.3.10  1.4.1.13  4.1.3.34  3.1.21.4  2.1.1.52  2.4.1.0  4.1.3.6  2.7.1.6  6.2.1.22  3.5.2.12  5.1.3.14  1.4.1.14  3.2.1.22  3.1.2.0  1.1.1.284  4.1.1.0  2.2.1.2  3.1.1.1  3.4.11.4  2.7.1.148 |
