# Supplementary material for: Genetic variability of mutans streptococci revealed by wide whole-genome sequencing
Source: BMC Genomics. 2013 Jun 28;14:430. doi: 10.1186/1471-2164-14-430 (PMC3751929; doi:10.1186/1471-2164-14-430)

To verify the unique presence of the lactate oxidase (consecutive) genes *sob|11000029* and *sob|11000030* respectively and to exclude the possibility of contamination with e. g. human DNA during the process of genome sequencing, PCR amplification with original DNA from *S. sobrinus* DSM 20742 & AC 153 and from strains *S. mutans* UA159 as well as *S. ratti* DSM 20564 (as negative controls) using one primer pair covering both genes was performed. The primers used were: 5’- GAGCAGGATAATTGACAGTC -3’ (forward primer), 5’- ACTCAGTGACGAATCAGTT -3’ (reverse primer), which were designed by using Primer Premier <http://www.premierbiosoft.com/primerdesign/index.html>) and Vector NTI 9.0 (InforMax) respectively. Conditions for this conventional PCR were: 94 °C, 2 min; followed by 32 cycles of 94 °C for 30s; annealing temperature 48°C for 30s; and 72 °C for 90s; final extension at 72°C for 5 min; length of amplicon 1,175 bp.


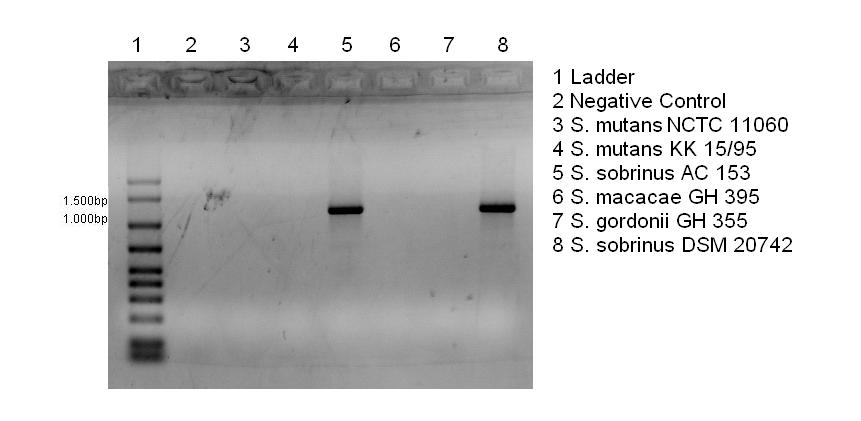

Supplement: Additional file 8 — PCR verifications of the unique presence of the lactate oxidase genes in S. sobrinus DSM 20742. [file 1471-2164-14-430-S8.docx]
