# Supplementary material for: Genetic variability of mutans streptococci revealed by wide whole-genome sequencing
Source: BMC Genomics. 2013 Jun 28;14:430. doi: 10.1186/1471-2164-14-430 (PMC3751929; doi:10.1186/1471-2164-14-430)
Supplement: Additional file 9 — The locations of missing genes in NCBI genome annotation results. [file 1471-2164-14-430-S9.docx]

## Location of *comS* in D816 (*S. mutans* 5DC8):

D816_00277 Contig001 62610-62663

## Location of *comS* in D817 (*S. mutans* KK21):

D817_00297 Contig001 67319-67372

## Location of *comS* in D818 (*S. mutans* KK23):

D818_00297 Contig003 40841-40894

## Location of *comS* in D819 (*S. mutans* AC4446):

D819_00203 Contig001 44940-44993

## Location of *comS* in D820 (*S. mutans* ATCC 25175):

D820_00247 Contig001 58302-58355

## Location of *comS* in D821 (*S. mutans* NCTC 11060):

D821_00253 Contig001 58943-58996

## Location of *comS* in D822 (*S. ratti* DSM 564):

D822_01077 Contig029 24624-24571

## Location of D819_02786 in D819 (*S. mutans* AC4446):

D819_02786 Contig016 116895-117830

## Location of D823_06595 in D823 (*S. sobrinus* DSM 20742):

D823_06595 Contig105 23045-22380

## Location of D823_06598 in D823 (*S. sobrinus* DSM 20742):

D823_06598 Contig105 23482-23042
